# TSLP in DRG neurons causes the development of neuropathic pain through T cells

**DOI:** 10.1186/s12974-023-02882-y

**Published:** 2023-09-02

**Authors:** Yuka Ino, Motoyo Maruyama, Masumi Shimizu, Rimpei Morita, Atsuhiro Sakamoto, Hidenori Suzuki, Atsushi Sakai

**Affiliations:** 1 Department of Anesthesiology, Nippon Medical School, 1-1-5 Sendagi, Bunkyo-ku, Tokyo, 113-8602 Japan; 2 Department of Pharmacology, Nippon Medical School, 1-1-5 Sendagi, Bunkyo-ku, Tokyo, 113-8602 Japan; 3 Division of Laboratory Animal Science, Nippon Medical School, 1-1-5 Sendagi, Bunkyo-ku, Tokyo, 113-8602 Japan; 4 Department of Microbiology and Immunology, Nippon Medical School, 1-1-5 Sendagi, Bunkyo-ku, Tokyo, 113-8602 Japan

**Keywords:** Neuropathic pain, Thymic stromal lymphopoietin, Dorsal root ganglion, T cell, Cathepsin S, IL-24, CXCL13, Infant

## Abstract

**Background:**

Peripheral nerve injury to dorsal root ganglion (DRG) neurons develops intractable neuropathic pain via induction of neuroinflammation. However, neuropathic pain is rare in the early life of rodents. Here, we aimed to identify a novel therapeutic target for neuropathic pain in adults by comprehensively analyzing the difference of gene expression changes between infant and adult rats after nerve injury.

**Methods:**

A neuropathic pain model was produced in neonatal and young adult rats by spared nerve injury. Nerve injury-induced gene expression changes in the dorsal root ganglion (DRG) were examined using RNA sequencing. Thymic stromal lymphopoietin (TSLP) and its siRNA were intrathecally injected. T cells were examined using immunofluorescence and were reduced by systemic administration of FTY720.

**Results:**

Differences in changes in the transcriptome in injured DRG between infant and adult rats were most associated with immunological functions. Notably, TSLP was markedly upregulated in DRG neurons in adult rats, but not in infant rats. TSLP caused mechanical allodynia in adult rats, whereas TSLP knockdown suppressed the development of neuropathic pain. TSLP promoted the infiltration of T cells into the injured DRG and organized the expressions of multiple factors that regulate T cells. Accordingly, TSLP caused mechanical allodynia through T cells in the DRG.

**Conclusion:**

This study demonstrated that TSLP is causally involved in the development of neuropathic pain through T cell recruitment.

**Supplementary Information:**

The online version contains supplementary material available at 10.1186/s12974-023-02882-y.

## Background

Damage to the somatosensory system often causes chronic neuropathic pain, but current analgesics are often hampered by insufficient efficacy and adverse effects. However, neuropathic pain is absent in the early life of rodents with peripheral nerve injury [1–4], although the specific prevalence of neuropathic pain in children is unknown [5]. Glial responses, including inflammatory gene expression in the spinal cord, were different between adult and infant rats after peripheral nerve injury [6]. Both spinal and peripheral neuroinflammation are a major cause of neuropathic pain [7, 8]. In the spinal cord, weak microglial activation following infant nerve injury was accompanied by a distinct dorsal horn cytokine response that differed considerably from that following adult nerve injury [9]. SIRT1, an aging-related protein, in dorsal root ganglion (DRG) neurons was suggested to be related to differential neuropathic pain vulnerability in adult and juvenile rodents [10

Thymic stromal lymphopoietin (TSLP) is an epithelial cell-derived cytokine that contributes to allergic inflammation, including atopic dermatitis and asthma [11]. In asthma, TSLP is increased in the airways of humans and rodents [12], and an anti-TSLP monoclonal antibody, tezepelumab, was effective and safe for the treatment of severe asthma in a phase 3 clinical trial [13]. TSLP mediates its effects on various immune cells through heterodimeric receptors composed of a unique TSLP receptor (TSLPR) and IL-7 receptor alpha chain (IL-7R). Signaling through these receptors induces the production of multiple cytokines [14]. In particular, T cell migration is stimulated by TSLP directly and indirectly through dendritic cells [11, 15]. T cells, especially CD4^+^ T cells, infiltrate the DRG in response to various types of nerve injury, and T helper cells are involved in the development of chronic tactile allodynia after nerve injury [16–18].

Here, we identified TSLP as a key regulator for the development of neuropathic pain by a comprehensive comparison of nerve injury-induced transcriptome changes in the DRG between adult and infant rats. TSLP was causally involved in the development of neuropathic pain and induced T cell recruitment into the DRG and upregulation of multiple factors that affect T cell functions. Therefore, anti-TSLP therapy may provide effective and safe medication for adult patients with neuropathic pain.

## Methods

### Animal models

All experimental procedures were approved by the President of the Nippon Medical School (approval number 27–037 and 2020-042) and were performed in accordance with the guidelines of the International Association for the Study of Pain [19]. Male and female Sprague–Dawley rats (Sankyo Labo Service Corporation, Tokyo, Japan) were used for all experiments. The rats were allowed free cage activity and food and water ad libitum. Infant rats were weaned 21 days after birth. For surgery, all rats were deeply anesthetized by the inhalation of 2–4% isoflurane (Pfizer Japan, Tokyo, Japan). Neuropathic pain was induced in neonatal (7 days old) and young adult (5–6 weeks old) rats by spared nerve injury (SNI), as previously described [20]. Briefly, the left (ipsilateral) sciatic nerve was exposed in the upper lateral thigh. The common peroneal and tibial branches of the sciatic nerve were tightly ligated with 4–0 silk thread and transected distally leaving the sural nerve intact. For sham surgery, the sciatic nerve was exposed but not ligated. The right (contralateral) sciatic nerve was left intact, and the right DRG was used as a control in the quantitative PCR (qPCR) and histological experiments. Results from both sexes were combined as no obvious sex differences were observed, although the study was not powered to study small sex differences.

### Behavioral tests

Paw withdrawal responses to mechanical stimuli were measured using a set of von Frey filaments (Muromachi Kikai, Tokyo, Japan). Each rat was placed on a metallic mesh floor covered with a plastic box. A von Frey filament was applied from underneath the mesh floor to the plantar surface of the hind paw. The weakest force that induced hind paw withdrawal at least three times in five trials was defined as the paw withdrawal threshold. The effects of TSLP or TSLP knockdown were examined by an investigator blinded to the experimental conditions.

### RNA sequencing

The lumbar fifth (L5) DRG was removed 14 days after SNI or sham surgery in adult and neonatal rats (*n* = 4). The removed DRG was frozen in liquid nitrogen and stored at − 80 °C until RNA purification. For RNA purification, total RNA was extracted using RNAiso Plus following the manufacturer’s instruction (Takara Bio, Shiga, Japan). RNA sequencing and data analysis were performed by GENEWIZ (South Plainfield, NJ). Poly-A RNA was prepared from 500 ng of total RNA using an NEBNext Poly (A) mRNA Magnetic Isolation Module (New England Biolabs, Ipswich, MA). A strand-specific cDNA library was synthesized using an NEBNext Ultra II Directional RNA Kit from Illumina (New England Biolabs) and was sequenced using an HiSeq SBS kit and HiSeq 4000 (Illumina, San Diego, CA). Raw read data were filtered using Cutadapt and the filtered read data were aligned to the rat genome (Rnor_6.0) using Hisat2 with default parameters. Gene differential analysis was performed using DESeq2. Bioinformatic analysis of the gene functions was performed using Ingenuity Pathway Analysis software (Qiagen, Redwood City, CA).

### qPCR

Total RNA (500 ng) was reverse-transcribed with a random primer by an iScript Select cDNA Synthesis kit (Bio-Rad, Hercules, CA). The reverse-transcription reaction consisted of 5 min at 25 °C, 30 min at 42 °C, and 5 min at 85 °C. qPCR solution was prepared using a Power SYBR Green PCR Master Mix (Thermo Fisher Scientific) and gene-specific primer pairs (Additional file 1: Table S1). The PCR was performed using a StepOnePlus Real-time PCR System (Thermo Fisher Scientific) at 95 °C for 10 min, followed by 40 cycles consisting of 95 °C for 15 s and 60 °C for 1 min. For IL-6, PCR was performed using a TaqMan Gene Expression Master Mix using a premix of a gene-specific TaqMan probe and primer pairs (Rn01410330; Thermo Fisher Scientific). The PCR program was initiated by 50 °C for 2 min and 95 °C for 10 min, followed by 40 cycles of 95 °C for 15 s and 60 °C for 1 min. All samples were measured in triplicate and the relative expression was calculated according to the 2^−ΔΔCT^ method.

### Drug administration

For intrathecal drug delivery, a polyethylene catheter (PE-10) filled with saline was inserted into the subarachnoid space between the cranial bone and the atlas 3 days before drug administration. The tip of the catheter was inserted to the level of the thoracic vertebrae. Rats with paralysis of a hind foot were excluded from the experiments. From 3 days before SNI surgery, 2 µg/10 µl of TSLP siRNA (Sigma-Aldrich, St. Louis, MO) or negative control siRNA (SIC-001) followed by 10 µl of saline (flush) was administered daily through the intrathecal catheter. TSLP siRNA sequences (SASI_Rn02_00321704) were 5′-AUGGGAUCUUGUUCGACCA-3′ (sense) and 5′-UGGUCGAACAAGAUCCCAU-3′ (antisense). Recombinant human TSLP (1 µg/10 µl; PeproTech, NJ) or vehicle was intrathecally administrated followed by 10 µl of saline (flush) in adult naïve rats. FTY720 (Selleck Chemicals, Houston, TX) was dissolved in saline and intraperitoneally administered for 9 consecutive days from 6 days before first TSLP injection (0.3 mg/kg per day). For a control, saline was intraperitoneally injected.

### Immunofluorescence

Rats were perfused transcardially with PBS (pH 7.4) followed by fresh 4% paraformaldehyde in PBS. L5 DRGs were post-fixed in the same fixative overnight and cryoprotected in 20% sucrose in PBS at 4 °C overnight. They were rapidly frozen in OCT compound (Sakura Finetek, Tokyo, Japan) using dry ice/acetone. L5 DRG was cut into 10-µm sections using a cryostat (Leica Microsystems, Wetzlar, Germany). The sections were pre-incubated in PBS containing 5% normal donkey serum and 0.3% Triton X-100 for 30 min, and incubated with a mouse anti-αβ T-cell receptor antibody (1:250 dilution; 554911, BD Biosciences, Franklin Lakes, NJ). After washing in PBS for 5 min three times, the sections were incubated with a secondary antibody labeled with Alexa Fluor 488 or 594 (1:1000; Thermo Fisher Scientific) at room temperature for 1 h. Images were captured by a high-resolution digital camera equipped with a computer (Olympus, Tokyo, Japan). The number of T cells was counted by an investigator blinded to the experimental conditions. TCRαβ-positive T cells per area (mm^2^) were counted for three DRG sections obtained from each rat using ImageJ software (version 1.52; National Institutes of Health, Bethesda, MD).

### In situ hybridization

To produce RNA probes for in situ hybridization, fragments of the TSLP, TSLPR, and IL-7 receptor nucleotide sequences were amplified from rat DRG-derived cDNA using the following primers: TSLP (forward, 5′-ATGGTTCTTTTCAGGTACCT-3′ and reverse, 5′-TCAAGATTGAATGCAGGAAA-3′), TSLPR (forward, 5′- ATGCGAGCTGTGACCTGGGC-3′ and reverse, 5′-TTCCTTCACCCTGCGCATCC-3′), and IL-7R (forward, 5′-TCCCCCTCTCTCATTCACTTG-3′ and reverse, 5′-TAGATCTCCATCCTGGGCATTG-3′). The amplicon was inserted into the pGEM-T Easy Vector (Promega, Madison, WI) and the plasmid was linearized with an NcoI restriction enzyme for in vitro transcription. A digoxigenin-labeled antisense RNA probe was synthesized using SP6 RNA polymerase (Sigma-Aldrich).

For in situ hybridization, DRG sections were treated with 2 µg/ml proteinase K for 5 min at 37 °C, followed by 4% paraformaldehyde/PBS for 20 min at room temperature. After washing, the sections were hybridized with a digoxigenin-labeled RNA probe in a hybridization buffer (50% formamide, 5 × saline-sodium citrate (SSC) pH 4.5, 1% sodium dodecyl sulfate, 50 µg/ml heparin sodium, and 50 µg/ml yeast RNA) at 65 °C overnight. Sections were washed with a first wash buffer (50% formamide, 5 × SSC pH 4.5, and 1% sodium dodecyl sulfate) at 65 °C for 30 min and then with a second wash buffer (50% formamide and 2 × SSC pH 4.5) at 65 °C for 30 min three times. After incubation with 0.5% blocking solution (Roche Diagnostics) for 1 h, sections were incubated with an alkaline phosphatase-conjugated anti-digoxigenin antibody (1:1000; Roche Diagnostics) at 4 °C overnight. The sections were washed with tris-buffered saline containing 0.1% Tween-20 and 2 mM levamisole for 20 min at room temperature three times followed by a pre-incubation buffer (100 mM NaCl, 50 mM MgCl_2_, 100 mM Tris–HCl pH 9.5, 0.1% Tween-20, and 0.48 mg/ml levamisole) for 5 min at room temperature. The sections were stained with BM-purple (Roche Diagnostics) at room temperature for 5 days. Images were captured using a high-resolution microscope equipped with a computer (Olympus, Tokyo, Japan). To measure the sizes of primary sensory neurons, six DRG sections obtained from individual rats were analyzed using ImageJ software from the manually drawn outline of primary sensory neurons. An investigator blinded to the experimental conditions performed the counts of positive and negative cells.

### Cell sorting

L4–6 DRGs were removed from SNI rats at day 7, cut into small pieces, and incubated in PBS containing 5 mg/ml collagenase A and 1 mg/ml Dispase II for 30 min at 37 ℃. The DRG cells were dissociated in ice-cold Ham’s F12 nutrient mixture containing 15% fetal bovine serum by gentle pipetting. After blocking in 5% mouse serum in PBS for 30 min at 4 ℃, the cells were incubated with a PE-conjugated mouse anti-αβ T-cell receptor antibody (10 ng/μl; 554914, BD Biosciences). TCRαβ-positive and -negative cells were obtained from 7-AAD-negative populations using a BD FACS Aria II cell sorter (BD Biosciences). The sorted cells were subjected to qPCR as described above, except for the reverse transcription reaction, which was performed using SuperScript IV VILO Master Mix (Thermo Fisher Scientific).

### Statistical analysis

Values are expressed as the mean ± standard error of the mean (SEM). SPSS software (IBM, Armonk, NY) and Kyplot (version 6, KyenceLab, Tokyo, Japan) were used for statistical analyses. Normality of data was assessed by the Shapiro–Wilk test. *P*-values < 0.05 were considered statistically significant. Paired *t*-test was used for normally distributed data sets. The Wilcoxon signed-rank sum test, Mann–Whitney *U*-test, and Steel–Dwass test or Bonferroni correction for multiple comparisons were used if normal distribution was rejected.

## Results

### Marked differences in transcriptomic changes in the DRG between adult and infant rats after nerve injury

**Fig. 1:**
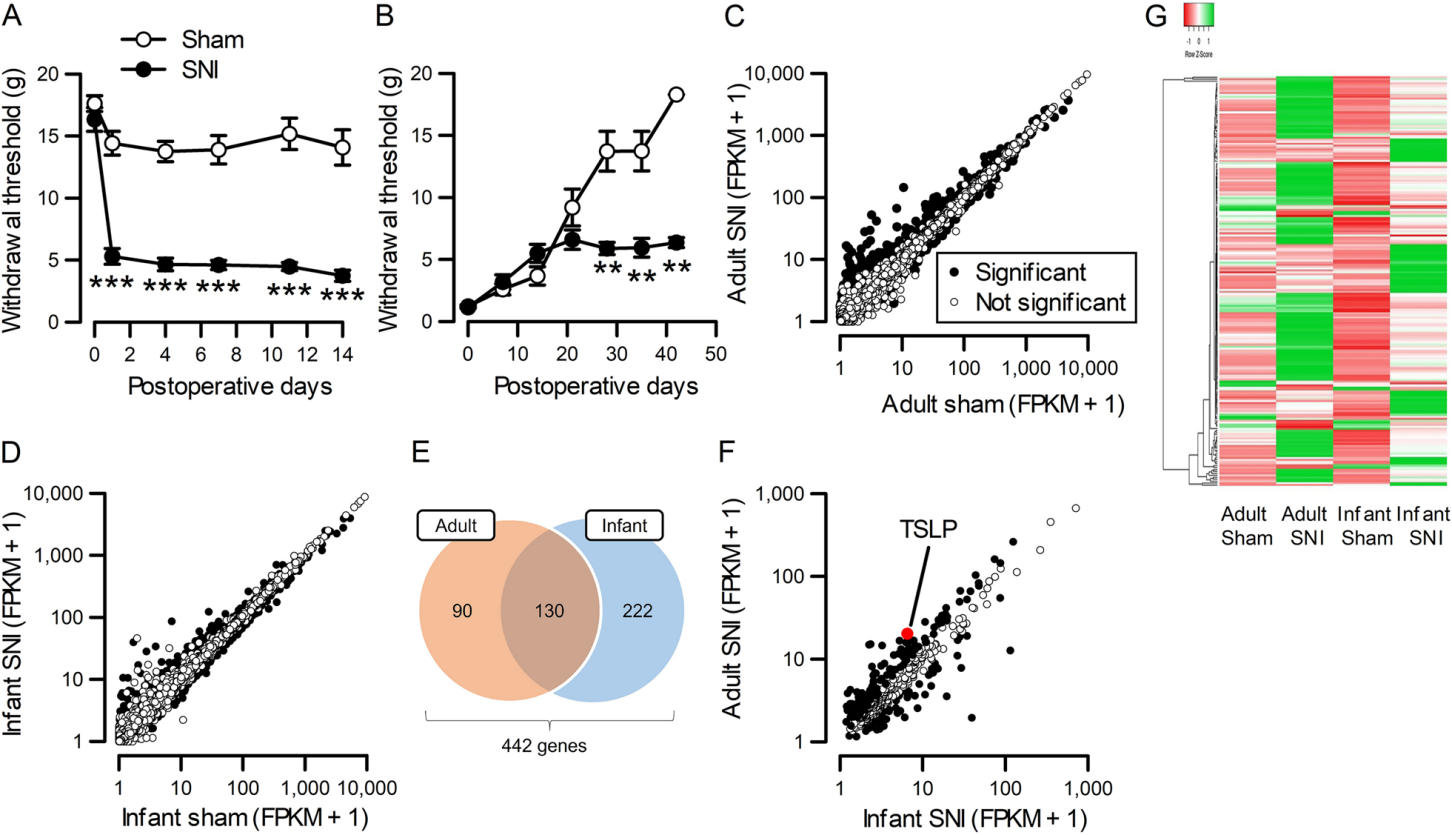
Difference in gene expression changes in the DRG of adult and infant rats after SNI. **A**, **B** Paw withdrawal thresholds were assessed after SNI or sham surgery in adult (**A**) and infant (**B**) rats (*n* = 6 males and 3 females). **P < 0.01 and ***P < 0.001 by Mann–Whitney *U*-test with Bonferroni correction. **C**, **D** RNA sequences were analyzed in the DRG 14 days after SNI or sham surgery in adult (**C**) and infant (**D**) rats (*n* = 4 males). Closed circles represent genes with statistically significant changes (*n* = 4). *q* < 0.05 by Benjamini–Hochberg adjusted p-value. **E** The Venn diagram shows the number of genes whose expression levels were significantly changed by more than two-fold in adult or infant rats. **F** Genes that were significantly changed after nerve injury in adult or infant rats were plotted. Closed circles represent genes with > 1.5 times differences in FPKMs between infant and adult SNI rats. **G** The heat map shows genes with > 1.5 times difference in FPKMs between infant and adult SNI rats

A neuropathic pain model induced by SNI, which transects the common peroneal and tibial nerves and ligates them to prevent regeneration, was produced on adult and neonatal rats because the degree of nerve injury remains unchanged as infant rats grow. SNI surgery on adult rats (5–6 weeks old) immediately and persistently decreased paw withdrawal thresholds compared with sham surgery (Fig. 1A), indicating the development of mechanical allodynia. In marked contrast, SNI surgery on neonatal rats (7 days old) did not decrease the paw withdrawal threshold for the first 3 weeks after surgery compared with sham surgery, although the thresholds normally increased in line with the growth of the rats (Fig. 1B). At 5 weeks of age, the paw withdrawal threshold of rats receiving neonatal SNI was significantly decreased compared with sham surgery, suggesting the development of mechanical allodynia. Thus, nerve injury in infant rats did not induce neuropathic pain behavior until adolescence, as previously described [21].

To identify genes undergoing differential expression changes between adult and infant rats, whole transcriptomic changes induced by peripheral nerve injury were examined in adult and infant rats receiving SNI or sham surgery. RNA sequencing was performed on L5 DRG of infant and adult rats 14 days after SNI or sham surgery, when mechanical allodynia had yet not developed in infant SNI rats. In adult and infant rats, SNI caused significant changes in gene expression in the DRG (Fig. 1C, D). Overall, 14,273 genes were detected with an FPKM of > 1 in one or more of the four groups. Among them, the expression levels of 220 and 352 genes in adult and infant rats, respectively, were significantly changed by more than two-fold. The number of genes changed in both adult and infant rats after nerve injury was 130, resulting in a total of 442 genes changed in adult or infant rats (Fig. 1E). To extract potential genes responsible for infant resistance to neuropathic pain from these 442 genes, the gene expression levels after SNI were compared between adult and infant rats (Fig. 1F). We found that 210 genes had > 1.5 times difference in expression levels between infant and adult SNI rats (Fig. 1G, Additional file 1: Table S2). These genes included known pro-nociceptive genes, including those encoding IL-6, neuronal nitric oxide synthase (nNOS), pituitary adenylate cyclase-activating peptide (PACAP), cathepsin S, CCL17, and CSF1, whose expression levels were higher in the adult SNI group compared with the infant SNI group (Additional file 1: Fig. S1A).

### Immunological response is the most distinct biofunction of DRG between adult and infant rats after nerve injury

**Fig. 2:**
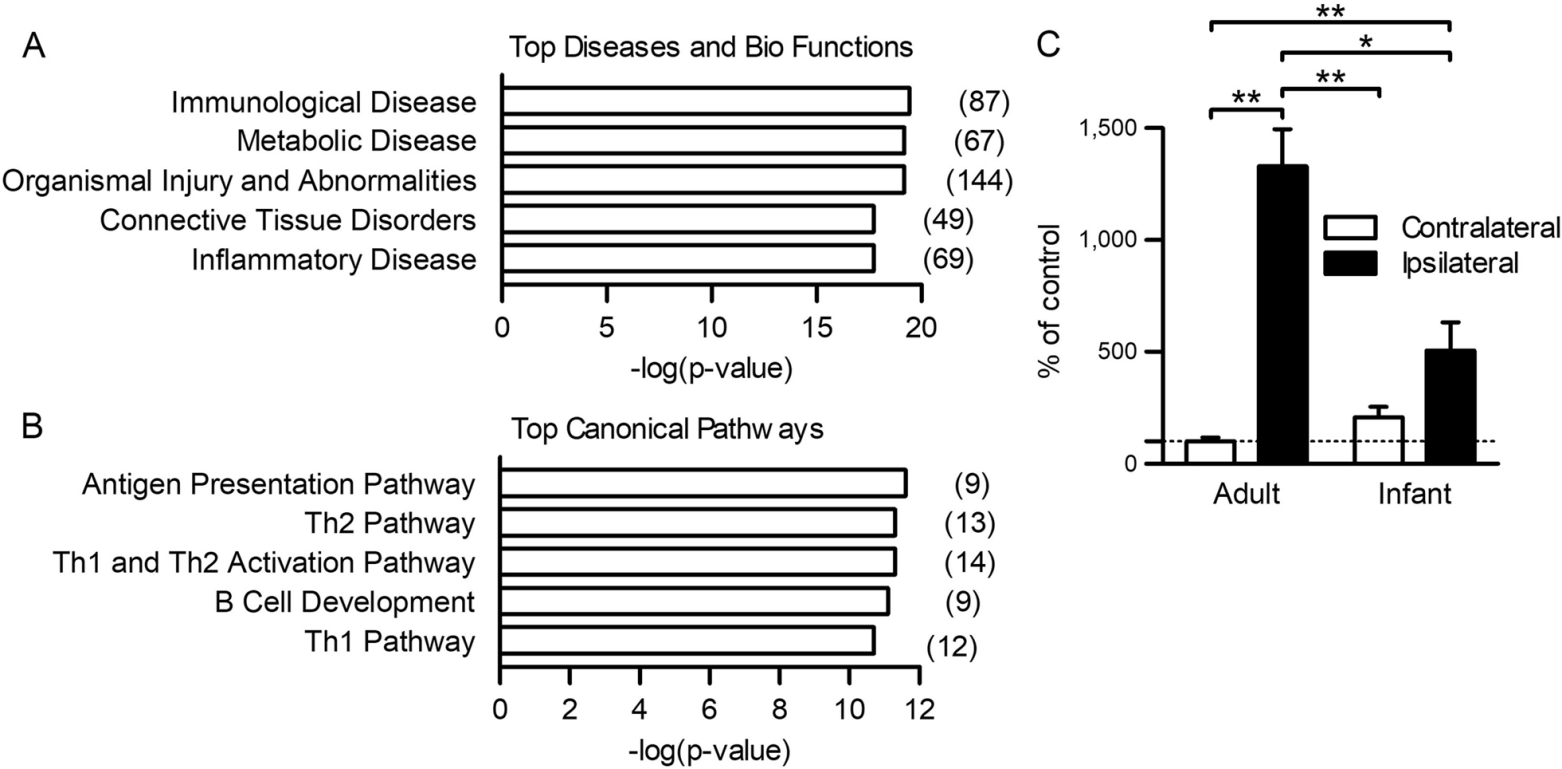
Bioinformatics analysis of differently expressed genes in the DRG between adult and infant SNI rats. **A**, **B** The list shows the top five diseases and bio functions (**A**) and the top five canonical pathways (**B**) related to genes with different expression levels in the DRG between adult and infant SNI rats. **C** Levels of TSLP were examined in the L5 DRG 14 days after SNI in adult (*n* = 6 males and 3 females) and infant (*n* = 6 males) rats. *P < 0.05 and **P < 0.01 by Steel–Dwass test

To further explore the molecular mechanisms responsible for infant resistance to neuropathic pain, bioinformatics analysis of the 210 genes with different expression levels in adult and infant SNI rats was performed using IPA. Gene ontology analysis showed that “immunological disease” was the most relevant in the “Diseases and Disorders” group (Fig. 2A) and involved 87 genes (Additional file 1: Table S3). In addition, “inflammatory response”, which involved 69 genes such as cytokines (Additional file 1: Table S4), was also related to the genes with differential expressions (Additional file 1: Table S5). Similarly, pathway analysis identified immune-related pathways, including T cell-related pathways, as Top Canonical Pathways (Fig. 2B). Because the proinflammatory immune response has a critical role in the pathophysiology of neuropathic pain in adult rodents [8], we focused on the immune-related genes that showed differential expression levels between adult and infant SNI rats. Among them, we found that TSLP was highly expressed in adult SNI compared with infant SNI, similar to IL-6 (Fig. 1F and Additional file 1: Tables S3, S4, S5). TSLP, a cytokine produced in response to environmental and inflammatory stimuli, is involved in inflammatory diseases and cancer [11]. qPCR confirmed that expression of TSLP in the L5 DRG of adult rats after SNI was markedly upregulated compared with infant rats (Fig. 2C and Additional file 1: Fig. S1B). TSLP expression in the contralateral DRG was not affected by SNI compared with the DRG of naïve rats (Additional file 1: Fig. S1C).

### TSLP expression is markedly induced in DRG neurons early after nerve injury

**Fig. 3:**
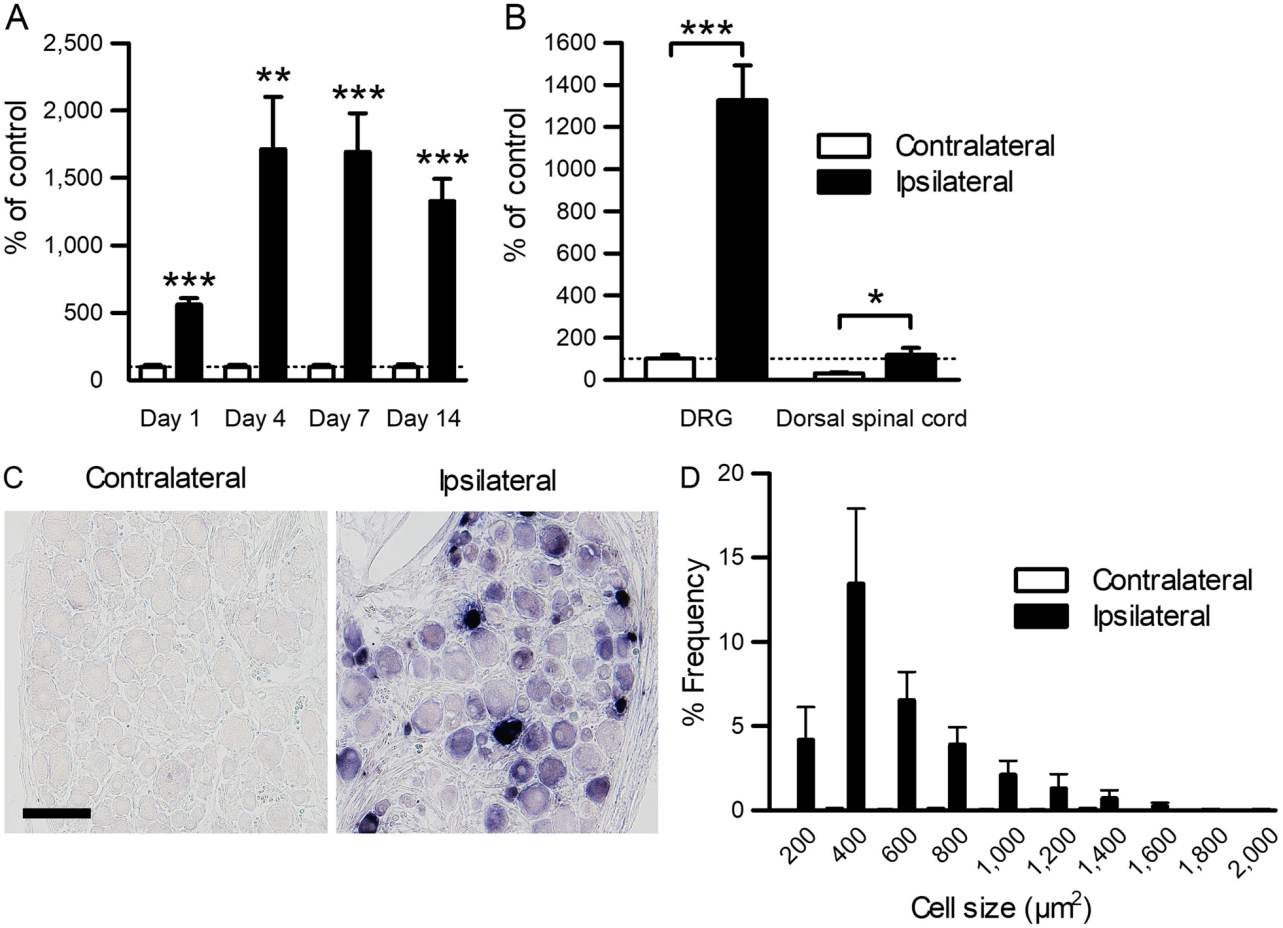
Expression changes in TSLP in the DRG neurons of adult rats after SNI. **A** Time course of TSLP expression changes after SNI was examined in the L5 DRG on the contralateral control and ipsilateral SNI sides using qPCR (*n* = 6 males and 3 females). **P < 0.01 and ***P < 0.001 by paired* t*-test. **B** Expression levels of TSLP in the adult L5 DRG (same dataset as (A); *n* = 6 males and 3 females) and dorsal spinal cord (*n* = 6 males) on the contralateral control and ipsilateral SNI sides 14 days after SNI. *P < 0.05 and ***P < 0.001 by paired *t*-test. **C** Representative in situ hybridization images of TSLP in the L5 DRG on the contralateral control and ipsilateral SNI sides 7 days after SNI. Scale bar = 100 µm. **D** Size distribution of TSLP-positive L5 DRG neurons (*n* = 4 male rats)

To investigate the involvement of TSLP in neuropathic pain, the time course of expression changes in adult rats was examined using qPCR. TSLP expression was increased from day 1 after nerve injury and remained increased until at least day 14 (Fig. 3A). The expression level of TSLP was also increased in the L5 dorsal spinal cord, although the basal expression level and the extent of change were much lower than those in the DRG (Fig. 3B). To identify the cell type expressing TSLP in the DRG, in situ hybridization was performed. Although only a weak signal of TSLP was observed in the control DRG, a strong signal was observed in many small and medium DRG neurons 7 days after nerve injury (Fig. 3C, D).

### TSLP contributes to the development of neuropathic pain

**Fig. 4:**
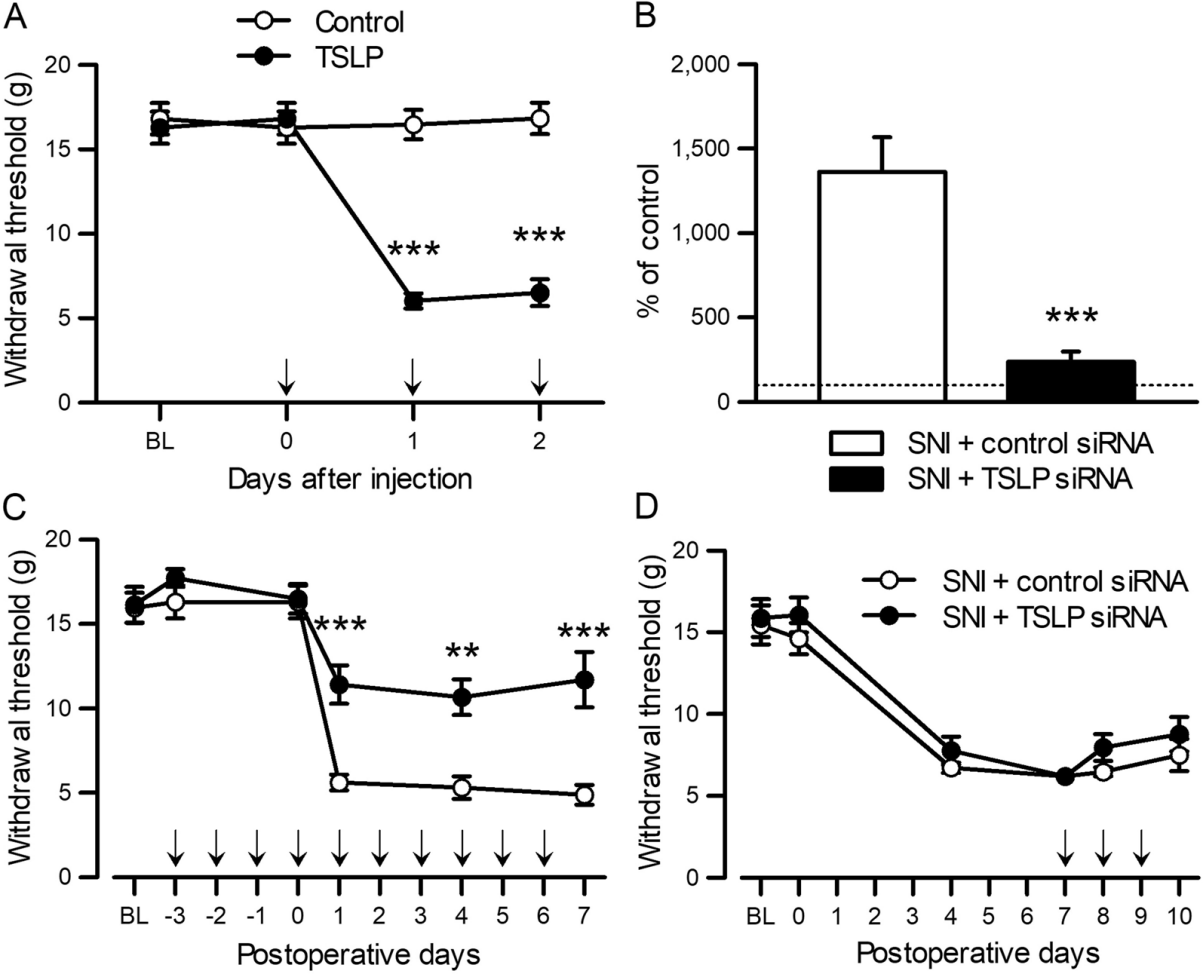
Suppression of TSLP prevents the development of neuropathic pain. **A** Paw withdrawal thresholds were examined before catheter implantation (BL), and before (day 0) and after intrathecal injection (*n* = 5 males and 3 females). ***P < 0.001 by Mann–Whitney *U-*test with Bonferroni correction. TSLP or vehicle was injected to adult naïve rats once a day from day 0 to day 2. **B** Expression levels of TSLP in the L5 DRG were examined 1 day after the last injection of siRNA by qPCR. TSLP siRNA or control siRNA was injected once a day from 3 days before SNI to 6 days after SNI. Values are expressed as a percentage of values for the control DRG as indicated by the dashed line (*n* = 6 males). ***P < 0.001 by unpaired *t*-test. **C**, **D** Paw withdrawal thresholds were examined in SNI rats receiving an intrathecal injection of TSLP siRNA or control siRNA before (**C**; *n* = 6 males and 2 females) and after (**D**; *n* = 6 males) SNI. siRNAs were injected once a day, as indicated by arrows. **P < 0.01 and ***P < 0.001 by Mann–Whitney *U*-test with Bonferroni correction

To investigate the role of TSLP in the modulation of nociception, recombinant TSLP was intrathecally injected into adult naïve rats once a day for 3 days. TSLP induced mechanical allodynia, which was sustained for more than 2 days (Fig. 4A). Next, to address the role of TSLP in the development and maintenance of neuropathic pain, TSLP siRNA was intrathecally injected before or after SNI. The increase of TSLP expression in the L5 DRG after nerve injury was significantly blocked by the intrathecal injection of TSLP siRNA (Fig. 4B and Additional file 1: Fig. S2). The administration of TSLP siRNA to intact adult rats did not affect the basal withdrawal threshold to mechanical stimuli at day 0 (Fig. 4C). However, the pre-emptive injection of TSLP siRNA suppressed the development of mechanical allodynia after SNI (Fig. 4C). In marked contrast, mechanical allodynia was not alleviated by TSLP knockdown after it had already developed (Fig. 4D). Therefore, TSLP was specifically involved in the development, but not maintenance, of mechanical allodynia.

### TSLP receptor subunits are expressed in DRG neurons and T cells

**Fig. 5:**
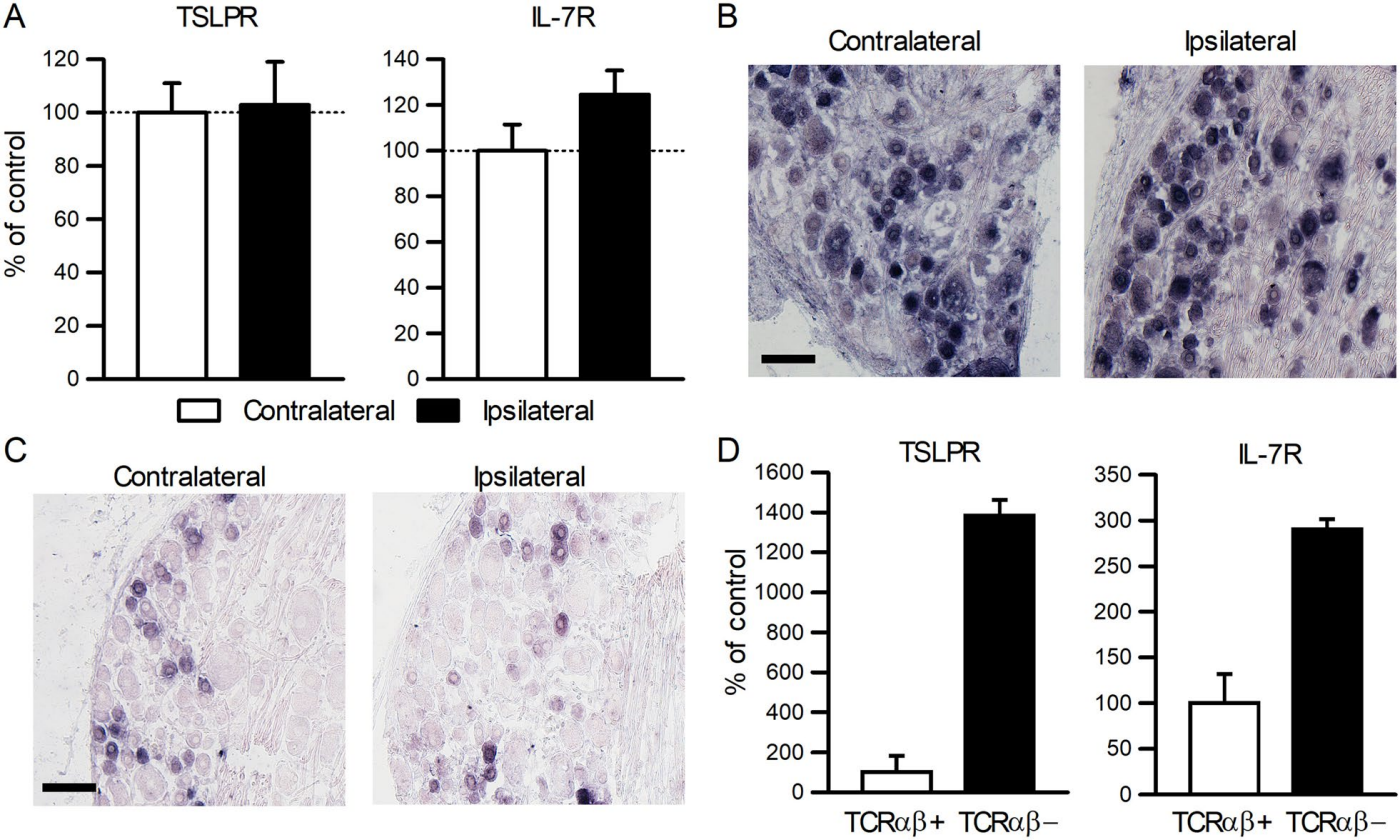
Expression of TSLP receptors, TSLPR and IL-7R, in the DRG. **A** Expression levels of TSLPR and IL-7R were examined in the L5 DRG 7 days after SNI (*n* = 6 males and 3 females). **B**, **C** Representative in situ hybridization images of TSLPR (**B**) and IL-7R (**C**) in the L5 DRG on the contralateral control and ipsilateral SNI sides 7 days after SNI (*n* = 4 male rats). Scale bars = 100 µm. **D** TSLPR and IL-7R expressions relative to TCRαβ-positive cells were examined by qPCR. TCRαβ-positive and -negative cells were sorted from L4 to 6 DRGs 7 days after SNI (*n* = 3 males)

Bioinformatic analysis identified T cell-related pathways as the Top Canonical Pathways (Fig. 2B). T cells have important roles in the development of neuropathic pain [16–18]. Consistent with this, TSLP was shown to regulate T cells directly and indirectly through non-T cells, including monocytes and dendritic cells [11, 15]. Therefore, we examined the expressions of the TSLP receptor subunits, TSLPR and IL-7R, in the DRG. The expression levels of TSLPR and IL-7R were not altered in the DRG after SNI (Fig. 5A). In situ hybridization showed that TSLPR and IL-7R were expressed in DRG neurons (Fig. 5B, C) as previously described [22]. However, the expressions of TSLPR and IL-7R could not be detected in other cell populations, which might be related to methodological limitations. Then, we used cell sorting to determine whether the receptors were expressed by infiltrated T cells. TSLPR and IL-7R were detected in T cells collected from the L4–L6 DRGs, although the non-T cell fraction, possibly containing various cells, including DRG neurons, satellite cells, and macrophages, showed an abundant expression of both receptors (Fig. 5D).

### TSLP regulates T cell infiltration into the DRG after nerve injury

**Fig. 6:**
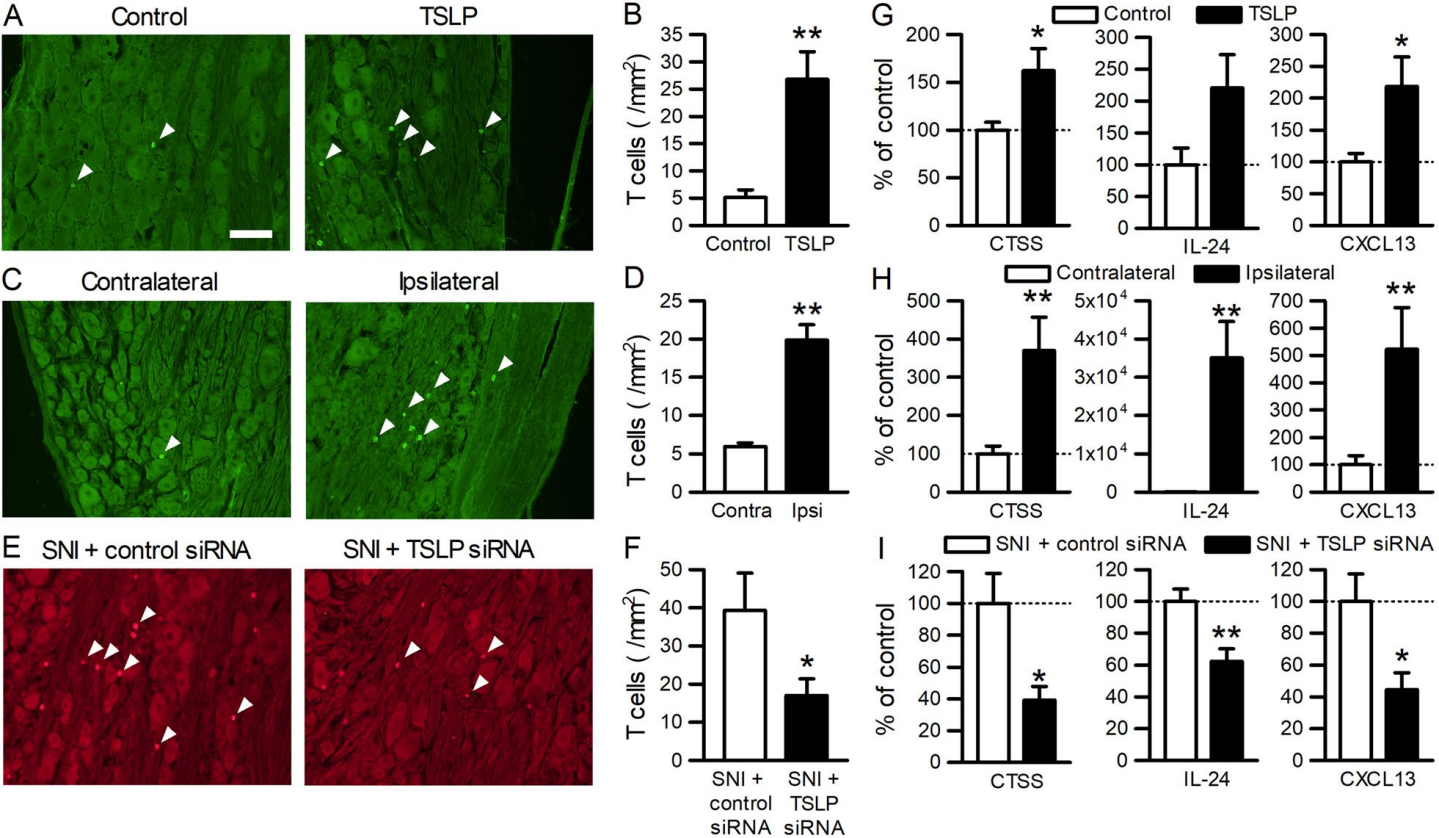
TSLP promotes T cell infiltration into the DRG in neuropathic pain. **A**, **C**, **E** Representative images of TCR-αβ immunofluorescence in the DRG of naïve rats administered TSLP (**A**), SNI rats (**C**), and SNI rats administered TSLP siRNA (**E**). Scale bar = 100 µm. **B**, **D**, **F** The number of T cells in the DRG was counted (*n* = 4 male rats). *P < 0.05 and **P < 0.01 by Mann–Whitney *U*-test. **G**–**I** Expression levels of cathepsin S, IL-24, and CXCL13 were examined in the L5 DRG of naïve rats administered TSLP (**G**; *n* = 6 males and 3 females), SNI rats (**H**; *n* = 6 males and 3 females), and SNI rats administered TSLP siRNA (**I**; *n* = 6 males and 2 females). *P < 0.05 and **P < 0.01 by unpaired *t*-test (**G**, **I**) or Wilcoxon signed-rank test (**H**). **A**, **B**, **G** DRGs were obtained 1 day after the intrathecal injection of TSLP for 3 consecutive days. **C**–**F**, **H**, **I** DRGs were obtained 7 days after SNI. TSLP siRNA or control siRNA was intrathecally injected in adult SNI rats from day − 3 to day 6

Next, we examined the effects of TSLP on the number of T cells because T cells infiltrate into the DRG after nerve injury [16]. The intrathecal injection of TSLP for 3 consecutive days significantly increased the number of T cells in the L5 DRG in adult naïve rats (Fig. 6A, B). Similarly, the number of T cells was increased in the L5 DRG 7 days after SNI (Fig. 6C, D). However, the increase in T cells following nerve injury was significantly blocked by the intrathecal injection of TSLP siRNA (Fig. 6E, F).

We further assessed the underlying mechanisms of T cell infiltration mediated by TSLP. Among the genes related to “immunological disease” (Additional file 1: Table S3) or “inflammatory response” (Additional file 1: Table S5) in the gene ontology analysis, nNOS, cathepsin S, IL-6, and IL-24 were reported to activate or recruit T cells [23–26]. In addition, CXCL13, which is a chemoattractant for a subset of T cells, was reported to be induced in the DRG after peripheral nerve injury [27], contributing to neuropathic pain [28]. Therefore, we examined the effect of TSLP on the expressions of these mediators. Expression levels of cathepsin S, IL-24, and CXCL13 were increased in the DRG after the intrathecal injection of TSLP (Fig. 6G). However, the expressions of nNOS and IL-6 were unaffected (Additional file 1: Fig. S3). Accordingly, cathepsin S, IL-24, and CXCL13 were upregulated in the DRG after nerve injury (Fig. 6H), and these increases were blocked by TSLP siRNA (Fig. 6I). Therefore, TSLP organized the early T cell infiltration into the DRG in neuropathic pain through multiple mechanisms.

### TSLP causes pain through T cell recruitment

**Fig. 7:**
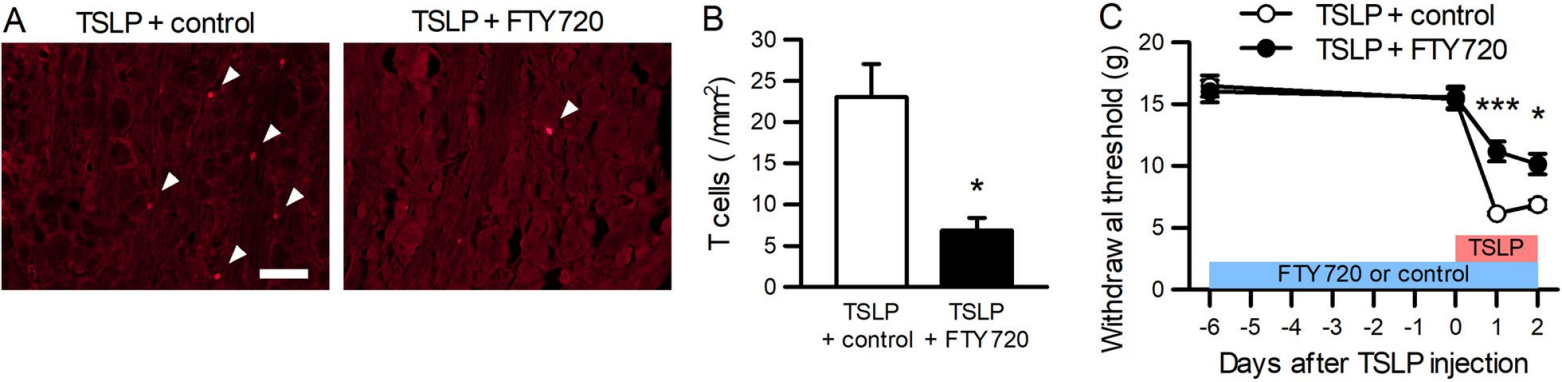
T cells mediate TSLP-induced mechanical allodynia. **A** Representative images of TCR-αβ immunofluorescence in the DRG of rats administered TSLP with FTY720 or saline. Scale bar = 100 µm. **B** The number of T cells in the DRG was counted (*n* = 4 male rats). *P < 0.05 by Mann–Whitney *U*-test. **C** Paw withdrawal thresholds were examined before FTY720 injection (day −6) and before (day 0) and after (days 1 and 2) TSLP injection. FTY720 (*n* = 5 males and 4 females) or saline (*n* = 6 males and 2 females) was intraperitoneally administered once a day for 9 consecutive days. TSLP was intrathecally injected once a day from day 0 to day 2. *P < 0.05 and ***P < 0.001 by Mann–Whitney *U*-test with Bonferroni correction

Involvement of T cells in TSLP-induced pain was examined using FTY720, a sphingosine-1-phosphate receptor agonist, which reduces the number of circulating T cells [29]. Pre-emptive administration of FTY720 to adult naïve rats suppressed the increase in T cells in the L5 DRG after intrathecal TSLP injection (Fig. 7A, B). FTY720 also suppressed mechanical allodynia induced by TSLP (Fig. 7C), although FTY720 alone did not affect basal paw withdrawal thresholds. Therefore, TSLP caused mechanical allodynia through T cells.

## Discussion

In this study, we identified TSLP as a critical regulator of the development of neuropathic pain through the analysis of infant resistance to neuropathic pain. A comprehensive comparison of nerve injury-induced gene expression changes revealed differential immunological and inflammatory processes in the DRG between infant and adult rats. Among them, TSLP was markedly upregulated in adult rats after nerve injury and was causally involved in the development of neuropathic pain. Mechanistically, TSLP induced T cell infiltration into the DRG through the expression of several genes that affect T cells. Consistent with this, TSLP caused pain behaviors through T cell recruitment. Therefore, TSLP may be a potential target for the prevention of neuropathic pain.

In this study, to identify novel targets for the effective treatment of neuropathic pain in adults, we examined the mechanisms underlying infant resistance to neuropathic pain, which has been observed in experimental animals [1, 2]. Although DRG neurons are primarily damaged in many neuropathic pain conditions, their involvement in infant resistance remained unclear. Through comprehensive gene expression analysis, we found that several key factors for neuropathic pain, including PACAP, cathepsin S, and nNOS [24, 30–33], were differentially regulated in the adult and infant DRGs after nerve injury. Furthermore, bioinformatics analysis revealed that a group of differentially regulated genes was closely related to immunological and inflammation responses, which are an important cellular basis for neuropathic pain [8]. In fact, expression levels of some cytokines (IL-6, TSLP, IL-24, and XCL1) were higher in adult SNI rats, while others (CCL11 and CXCL14) were lower, indicating a differential regulation of expression among distinct cytokines. Similarly, spinal inflammatory processes were suppressed in infant rats after peripheral nerve injury [6, 21, 34]. Because DRG neurons are a primary trigger for spinal neuroinflammation, differences in spinal inflammation in adult and infant rats may be at least partly attributed to suppressed proinflammatory responses in DRG neurons. In this regard, the expression level of CSF1, a well-known microglial activator of DRG neurons in neuropathic pain, was lower in infant than adult rats. Therefore, the characteristic features of infant DRG neurons involved in inflammatory responses, such as the weak induction of TSLP after nerve injury, might indicate therapeutic targets with high clinical relevance.

Changes in TSLP expression were markedly different in the adult and infant DRGs after nerve injury. The involvement of TSLP in neuropathic pain was unknown, although its increase was reported in DRG neurons after another type of peripheral nerve injury, chronic constriction injury of the sciatic nerve [22]. In this study, TSLP inhibition effectively prevented the development of neuropathic pain and increased the number of T cells after peripheral nerve injury. Consistent with this, TSLP was upregulated early after nerve injury and an intrathecal injection of TSLP caused pain behavior and increased T cell numbers in adult naïve rats. In fact, reducing circulating T cells by FTY720 [29, 35] blocked TSLP-induced T cell recruitment into the DRG as well as mechanical allodynia. Although FTY720 was shown to induce an aberrant activation of NFAT1, AP1, and NF-κB [36], the analgesic effect of FTY720 would not be obtained from their activation because activation of NFAT1, AP1, or NF-κB is known to cause neuropathic pain [37–39]. Furthermore, T cells in the injured DRG contributed to the development of neuropathic pain, although an anti-hyperalgesic role of T cells has also been described [16]. Interestingly, because T cell infiltration into the spinal cord is also suppressed in infant rats after peripheral nerve injury [40], TSLP derived from DRG neurons may be also involved in spinal T cell infiltration. Therefore, TSLP contributed to the induction of neuropathic pain through T cell recruitment into the DRG. However, because TSLP was increased in the dorsal spinal cord and intrathecal TSLP siRNA could suppressed the TSLP expression in spinal cells, spinal TSLP may be also involved in neuropathic pain and T cell infiltration.

TSLP was extensively induced in DRG neurons after nerve injury and regulated T cells, possibly through direct and/or indirect actions during the onset of neuropathic pain. Consistent with this, TSLP was reported to regulate T cells directly and indirectly via dendritic cells [11, 15]. Although their expression levels were unaffected by nerve injury, the TSLP receptor subunits (TSLPR and IL-7R) were also observed in DRG neurons, consistent with a previous report [22]. Indeed, TSLP derived from keratinocytes activated a subset of TRPA1-positive DRG neurons to induce itch sensations in atopic dermatitis [41], although intrathecal injection of TSLP did not show apparent scratching behaviors or skin ulcer 1 day after injection. Thus, TSLP released from DRG neurons might act on neighboring DRG neurons in an autocrine or paracrine manner and subsequently induce T cell infiltration. In line with this, TSLP indirectly activated T cells by regulating dendritic cell functions. TSLP induces the production of chemokines in dendritic cells through multiple intracellular signaling pathways, including STAT5/6, ERK, JNK, and NF-κB. The phosphorylation of JAK1 and JAK2 also induces and mediates the multiple biological functions of TSLP. In neuropathic pain, we found that TSLP upregulated several inflammatory genes known to activate T cells (cathepsin S, IL-24, and CXCL13). In the periphery, cathepsin S, a cysteine protease, released from macrophages [42] activated PAR2 in DRG neurons [43]. The inhibition of peripheral cathepsin S reversed T cell responses and neuropathic pain [44]. IL-24 is produced by activated monocytes, T cells, B cells, NK cells, and macrophages and promotes the production of proinflammatory cytokines in these peripheral blood mononuclear cells [45]. IL-24 promoted CD4^+^ T cell proliferation and activity [23], although its involvement in nociceptive modulation remains unknown [46]. CXCL13 is expressed in sensory neurons, such as trigeminal neurons [27, 47], and T cells [48–50]. The CXCL13 receptor, CXCR5, is expressed in a subset of T cells and B cells, which are attracted by CXCL13 [51, 52]. CXCR5 expression was shown in sensory neurons and astrocytes, and the inhibition of CXCL13 alleviated neuropathic pain [27, 47, 53]. Therefore, TSLP might promote T cell infiltration through the induction of several T cell regulators. However, TSLP receptors were also detected on T cells obtained from injured DRGs, suggesting the direct action of TSLP on T cells. Consistent with this, TSLP receptors are expressed in T cells [15, 54]. Through direct action on T cells, TSLP was reported to have a role in the maintenance of CD4^+^ T cell homeostasis and allergic skin inflammation [11]. Therefore, neuronal TSLP likely regulated T cell infiltration and functions through multiple pathways in neuropathic pain.

## Conclusions

TSLP is critically involved in T cell recruitment into the DRG during the development of neuropathic pain. Accordingly, TSLP upregulated multiple factors that regulate T cell infiltration and function. Given the efficacy and safety of an anti-TSLP monoclonal antibody, tezepelumab, in a phase 3 clinical trial of severe asthma [13] and a phase 2a clinical trial of atopic dermatitis [55], anti-TSLP therapy might also be a potential treatment for the development of neuropathic pain in adults.

### Supplementary Information

**Additional file 1: Figure S1.** FPKM of pro-nociceptive genes whose expression levels were higher in the adult SNI compared with infant SNI. (A) FPKM of PACAP, CCL17, CSF-1, IL-6, nNOS and cathepsin S in the L5 DRG 14 days after SNI or sham surgery in adult and infant rats (*n* = 4). (B) FPKM of TSLP in the L5 DRG 14 days after SNI or sham surgery in adult and infant rats (*n* = 4). (C) TSLP expression was compared between the contralateral DRG at day 7 after SNI and DRG of naïve rats (*n* = 5). **Figure S2.** Decreased expression of TSLP in the DRG neurons after intrathecal administration of TSLP siRNA. Representative in situ hybridization images of TSLP in the L5 DRG on the ipsilateral SNI sides 7 days after SNI. TSLP siRNA or control siRNA was injected once a day from 3 days before SNI to 6 days after SNI (*n* = 4 rats). Scale bar = 100 μm. **Figure S3.** Expression levels of nNOS and IL-6 were unaffected. Expression levels of nNOS and IL-6 in the L5 DRG after intrathecal injection of TSLP or vehicle once a day for 3 days (*n* = 3–6). **Table S1.** Primer pairs for quantitative PCR. **Table S2.** Differentially expressed genes more than 1.5 times after SNI at either developmental stage. **Table S3.** Genes involved in immunological diseases. **Table S4.** Cytokines involved in immunological diseases or inflammatory response. **Table S5.** Genes involved in inflammatory response.

## Data Availability

The datasets used and/or analysed during the current study are available from the corresponding author on reasonable request. The data of RNA sequencing are deposited to the GEO database (accession number GSE237869).

## Abbreviations

DRG: Dorsal root ganglion
IL-7R: IL-7 receptor alpha chain
L5: Lumbar fifth
nNOS: Neuronal nitric oxide synthase
PACAP: Pituitary adenylate cyclase-activating peptide (PACAP)
qPCR: Quantitative PCR
SNI: Spared nerve injury
SSC: Saline-sodium citrate
TSLP: Thymic stromal lymphopoietin
TSLPR: Thymic stromal lymphopoietin receptor
