# Supplementary material for: TSLP in DRG neurons causes the development of neuropathic pain through T cells
Source: J Neuroinflammation. 2023 Sep 2;20:200. doi: 10.1186/s12974-023-02882-y (PMC10474733; doi:10.1186/s12974-023-02882-y)
Supplement: Supplementary file 1 — Additional file 1: Figure S1. FPKM of pro-nociceptive genes whose expression levels were higher in the adult SNI compared with infant SNI. (A) FPKM of PACAP, CCL17, CSF-1, IL-6, nNOS and cathepsin S in the L5 DRG 14 days after SNI or sham surgery in adult and infant rats (n = 4). (B) FPKM of TSLP in the L5 DRG 14 days after SNI or sham surgery in adult and infant rats (n = 4). (C) TSLP expression was compared between the contralateral DRG at day 7 after SNI and DRG of naïve rats (n = 5). Figure S2. Decreased expression of TSLP in the DRG neurons after intrathecal administration of TSLP siRNA. Representative in situ hybridization images of TSLP in the L5 DRG on the ipsilateral SNI sides 7 days after SNI. TSLP siRNA or control siRNA was injected once a day from 3 days before SNI to 6 days after SNI (n = 4 rats). Scale bar = 100 μm. Figure S3. Expression levels of nNOS and IL-6 were unaffected. Expression levels of nNOS and IL-6 in the L5 DRG after intrathecal injection of TSLP or vehicle once a day for 3 days (n = 3–6). Table S1. Primer pairs for quantitative PCR. Table S2. Differentially expressed genes more than 1.5 times after SNI at either developmental stage. Table S3. Genes involved in immunological diseases. Table S4. Cytokines involved in immunological diseases or inflammatory response. Table S5. Genes involved in inflammatory response. [file 12974_2023_2882_MOESM1_ESM.pdf]

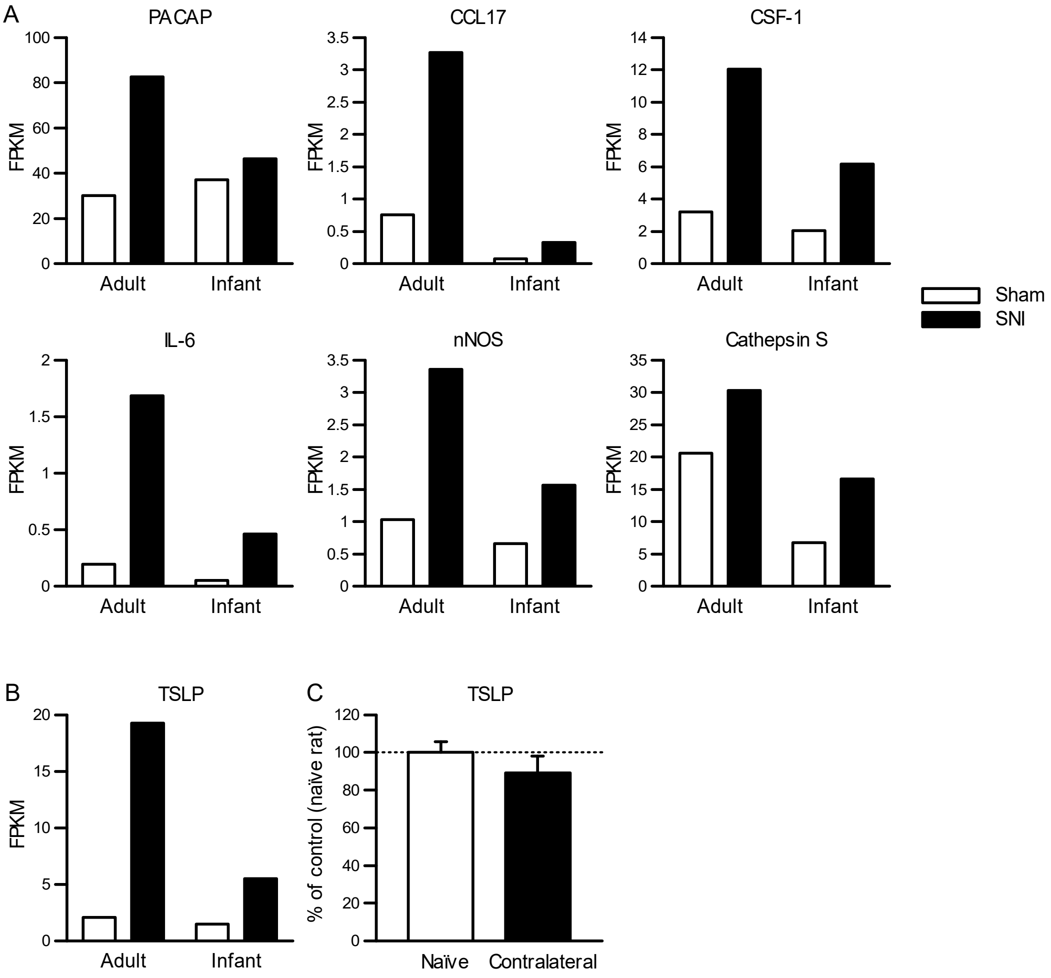

Figure S1. FPKM of pro-nociceptive genes whose expression levels were higher in the adult SNI compared with infant SNI.

(A) FPKM of PACAP, CCL17, CSF-1, IL6, NOS1 and CTSS in the L5 DRG 14 days after SNI or sham surgery in adult and infant rats (*n* = 4). (B) FPKM of TSLP in the L5 DRG 14 days after SNI or sham surgery in adult and infant rats (*n* = 4). (C) TSLP expression was compared between the contralateral DRG at day 7 after SNI and DRG of naïve rats (*n* =5).

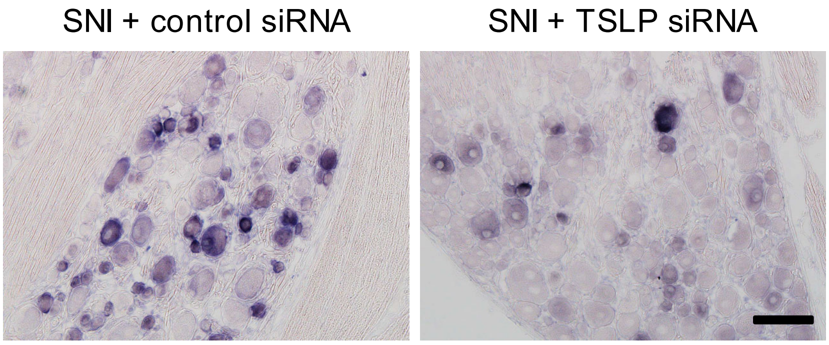

Figure S2. Decreased expression of TSLP in the DRG neurons after intrathecal administration of TSLP siRNA.

Representative in situ hybridization images of TSLP in the L5 DRG on the ipsilateral SNI sides 7 days after SNI. TSLP siRNA or control siRNA was injected once a day from 3 days before SNI to 6 days after SNI (*n* = 4 rats). Scale bar = 100 µm.

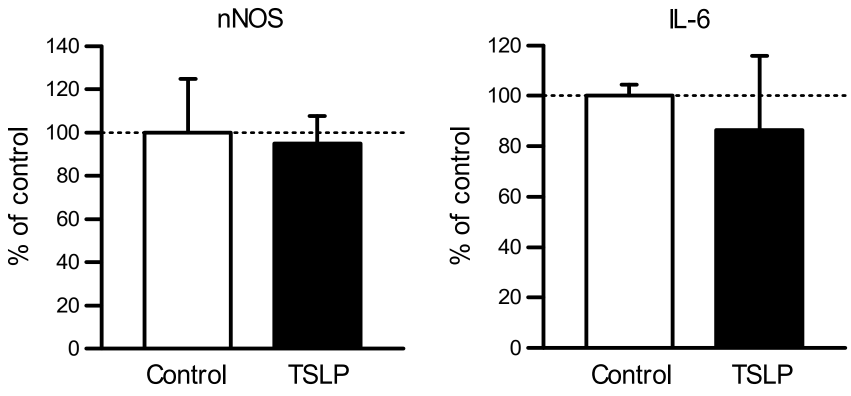

Figure S3. Expression levels of nNOS and IL-6 were unaffected. Expression levels of nNOS and IL-6 in the L5 DRG after intrathecal injection of TSLP or vehicle once a day for 3 days (n = 3–6).

Table S1

Primer paires for quantitative PCR

Gene	Forward	Reverse
TSLP	5´-CGAAAGCGGCCCTCATTAAC-3´	5´-AATGCAGGAAAGCCACAATCC-3´
TSLPR	5´-CGTGGCATCCCAATGAGC-3´	5´-GTCGCGAGTTCTAGGGTCG-3´
IL-7R	5´-TCCCCCTCTCTCATTCACTTG-3´	5´-TAGATCTCCATCCTGGGCATTG-3´
Cathepsin S	5´-CCACTAAAGGGCCTGTCTCT-3´	5´-CCCATAGCCAACCACGAGAA-3´
CXCL13	5´-GGCTGCCCCAAAACTGAAATC-3´	5´-AGTAATACTTCTGCTTCGGACAAA-3´
IL-24	5´-ACAAGTGTCCGGCTGTTGAA-3´	5´-TGTGGGCAAGGTAACAGCTC-3´
nNOS	5´-CCTTCCGAAGCTTCTGGCAACAGC-3´	5´-TGGACTCAGATCTAAGGCGGTTGG-3´

Table S2

Differentially expressed genes more than 1.5 times after SNI at either developmental stage.

Gene Symbol	Entrez Gene Name	FPKM (Adult Sham)	FPKM (Adult SNI)	padj (Adult)	Fold Change (Adult)	FPKM (Infant Sham)	FPKM (Infant SNI)	padj (Infant)	Fold Change (Infant)	FPKM (Adult SNI) /FPKM (Infant SNI)
AABR07003537.1		5.9875	12.185	0.0000259	2.035073069	4.54	7.0625	0.02469321	1.55561674	1.725309735
AABR07005593.1		0.12	1.3775	2.17E-21	11.47916667	0.0625	0.365	1.54059E-05	5.84	3.773972603
AABR07009834.1		23.1575	65.435	8.53E-24	2.825650437	23.5475	36.6725	2.77309E-10	1.557384011	1.784307042
AABR07013477.2		0.0825	1.37	9.03E-09	16.60606061	0.035	0.6275	6.20465E-07	17.92857143	2.183266932
AABR07025368.1		0.18	2.1075	3.33E-12	11.70833333	0.2475	0.665	0.02233727	2.686868687	3.169172932
AABR07040095.1		0.295	0.355	0.589442251	1.203389831	0.4325	1.0475	3.2681E-11	2.421965318	0.338902148
AABR07042633.1		0.125	2.895	6.77E-14	23.16	0.02	0.8375	9.38275E-09	41.875	3.456716418
AABR07044837.2		1.0625	10.62	1.11E-17	9.995294118	0.305	4.12	1.04689E-18	13.50819672	2.577669903
AABR07045485.1		0.8875	2.445	0.120209054	2.754929577	0.58	5.505	1.48049E-48	9.49137931	0.444141689
AABR07049695.2		1.12	1.0725	0.911017684	0.957589286	1.0925	2.2225	0.014947987	2.034324943	0.482564679
AABR07050265.1		2.05	0.7675	0.017434699	0.374390244	3.8475	1.7625	0.002537738	0.458089669	0.435460993
AABR07058423.4		1.545	0.75	0.086292261	0.485436893	0.7575	0.185	0.005233768	0.244224422	4.054054054
AABR07058464.1		3.8975	2.1725	NA	0.557408595	1.4425	3.75	0.004478184	2.59965338	0.579333333
AABR07070720.1		15.2225	5.74	0.0000578	0.377073411	24.45	11.56	7.50021E-06	0.472801636	0.496539792
AC111647.1		0.53	1.7225	0.01559209	3.25	0.5375	1.0025	0.170276508	1.865116279	1.718204489
Adcyap1	adenylate cyclase activating polypeptide	30.015	82.485	5.91E-11	2.748125937	37.075	46.32	0.027866661	1.249359407	1.780764249
Adgrd1	adhesion G protein-coupled receptor D1	1.3875	3.2	0.000000109	2.306306306	0.3825	1.3025	4.32412E-12	3.405228758	2.45681382
Agt	angiotensinogen	3.0925	1.8975	0.003895937	0.613581245	0.7675	0.3825	6.49122E-05	0.498371336	4.960784314
Angptl1	angiopoietin-like 1	0.7925	0.695	0.776133117	0.876971609	0.54	1.195	0.000724605	2.212962963	0.581589958
Ankrd1	ankyrin repeat domain 1	0.4075	5.36	2.41E-48	13.15337423	0.2425	1.5125	7.08489E-16	6.237113402	3.543801653
Arsi	arylsulfatase family member I	0.38	0.4575	0.799264376	1.203947368	0.515	1.22	0.003508219	2.368932039	0.375
Atf3	activating transcription factor 3	7.18	65.9475	2.46E-10	9.184888579	1.7725	27.425	3.65545E-19	15.47249647	2.404649043
Btc	betacellulin	0.9725	1.035	0.903427101	1.064267352	0.8125	1.8375	0.000559397	2.261538462	0.563265306
C1qa	complement C1q A chain	17.86	34.2025	5.72E-09	1.915033595	6.8825	17.74	7.85854E-06	2.577551762	1.927987599
C1qb	complement C1q B chain	13.6975	23.3	NA	1.701040336	4.805	12.745	4.63156E-05	2.652445369	1.828167909
C1qc	complement C1q C chain	15.3175	25.925	3.22E-11	1.692508569	6.375	14.515	0.001190112	2.276862745	1.786083362
Ccdc172	coiled-coil domain containing 172	3.8875	10.2175	7.74E-28	2.62829582	4.45	6.5475	0.001984488	1.471348315	1.560519282
Ccdc3	coiled-coil domain containing 3	2.5475	2.6225	0.988390298	1.029440628	1.5125	4.135	0.000616864	2.733884298	0.634220073
Cckbr	cholecystokinin B receptor	1.24	6.4825	0.000000139	5.227822581	0.45	3.4	0.00041335	7.555555556	1.906617647
Ccl11	C-C motif chemokine ligand 11	0.4075	0.665	0.322917373	1.63190184	0.4425	1.08	0.006398439	2.440677966	0.615740741
Ccl17	C-C motif chemokine ligand 17	0.755	3.2675	0.00000956	4.32781457	0.0725	0.3275	NA	4.517241379	9.977099237
Ccnd2	cyclin D2	4.7725	5.87	0.336986557	1.229963332	6.105	12.3375	1.97013E-10	2.020884521	0.475785208
Ccr5	C-C motif chemokine receptor 5	1.7475	2.61	0.388156054	1.493562232	0.6325	1.655	4.18957E-09	2.616600791	1.577039275
Cd68	CD68 molecule	3.5925	4.11	0.867529046	1.144050104	1.2625	2.535	0.00215456	2.007920792	1.621301775
Cd74	CD74 molecule	95.3925	261.31	3.7E-11	2.739313887	26.9525	122.905	3.42357E-08	4.560059364	2.126113665
Cd86	CD86 molecule	0.5925	1.1325	0.003145335	1.911392405	0.27	0.66	0.007006358	2.444444444	1.715909091
Cenpu	centromere protein U	0.4425	1.13	0.001513847	2.553672316	0.4625	0.72	0.100236624	1.556756757	1.569444444
Chrdl1	chordin like 1	0.9875	1.2575	0.503420853	1.273417722	0.2125	0.44	0.006958727	2.070588235	2.857954545
Cidea	cell death inducing DFFA like effector a	2.74	6.5725	0.000295126	2.398722628	2.2825	2.985	0.421733885	1.307776561	2.201842546
Ciita	class II major histocompatibility complex transactivator	0.7	1.58	1.71E-08	2.257142857	0.2675	0.5925	0.000406758	2.214953271	2.666666667
Ckmt2	creatine kinase, mitochondrial 2	2.8925	1.4925	0.001012659	0.515989628	2.3	0.6225	1.94871E-18	0.270652174	2.397590361
Cldn11	claudin 11	14.005	10.035	0.656155165	0.716529811	11.79	25.165	0.018347937	2.134435963	0.39876813
Cldn20	claudin 20	1.815	0.89	0.018719842	0.490358127	2.7425	1.3925	2.98076E-05	0.507748405	0.639138241
Clec10a	C-type lectin domain containing 10A	9.5225	12.6775	0.016124652	1.331320557	2.4025	5.7675	2.39947E-06	2.40062435	2.198092761
Clec12a	C-type lectin domain family 12 member A	4.015	6.465	0.005325214	1.610211706	1.555	3.64	0.000103762	2.340836013	1.776098901
Clec4a3	C-type lectin domain family 4, member a3	5.5775	7.0225	0.510673692	1.259076647	1.5025	4.335	0.001632251	2.885191348	1.619953864
Clec7a	C-type lectin domain containing 7A	1.9925	5.74	6.45E-14	2.880803011	0.365	1.7075	2.76548E-12	4.678082192	3.361639824
Col2a1	collagen type II alpha 1 chain	14.495	6.8325	0.002772913	0.471369438	47.415	28.19	1.67908E-07	0.594537594	0.242373182
Col8a1	collagen type VIII alpha 1 chain	5.245	6.6425	0.739325229	1.266444233	3.9325	11.7875	0.013957135	2.997457088	0.563520679
Crisp3	cysteine-rich secretory protein 3	1.495	27.2025	7.75E-16	18.19565217	0.165	9.655	3.51919E-12	58.51515152	2.817452097
Crlf1	cytokine receptor like factor 1	8.1325	14.915	0.025361465	1.833999385	1.595	5.2475	3.40395E-09	3.289968652	2.84230586
Cryba2	crystallin beta A2	4.95	15.8275	0.001432223	3.197474747	3.5275	6.7725	0.003822653	1.919914954	2.337024732
Crygs	crystallin gamma S	1.295	0.5775	0.014659254	0.445945946	0.3775	0.3475	0.843222123	0.920529801	1.661870504
Csf1	colony stimulating factor 1	3.205	12.0275	2.6E-34	3.752730109	2.055	6.15	2.02906E-09	2.99270073	1.955691057
Csrnp1	cysteine and serine rich nuclear protein 1	1.5675	2.8225	0.000131252	1.800637959	0.9125	1.87	0.000843378	2.049315068	1.509358289
Csrp3	cysteine and glycine rich protein 3	1.7175	16.81	3.02E-09	9.787481805	0.39	9.605	1.14832E-07	24.62820513	1.750130141
Cst7	cystatin F	0.74	1.02	0.794996428	1.378378378	0.07	0.37	0.000468599	5.285714286	2.756756757
Cthrc1	collagen triple helix repeat containing 1	2.095	2.6125	0.470644707	1.247016706	3.1275	6.995	3.657E-06	2.236610711	0.373481058
Ctss	cathepsin S	20.63	30.3075	0.002514994	1.4690984	6.735	16.575	0.00601047	2.461024499	1.828506787
Cxadr	CXADR Ig-like cell adhesion molecule	8.805	15.995	0.007915178	1.816581488	10.84	25.36	2.01467E-05	2.339483395	0.630717666
Cxcl14	C-X-C motif chemokine ligand 14	0.39	1.2425	0.000609982	3.185897436	0.355	3.925	1.37058E-18	11.05633803	0.31656051
Cyp2s1	cytochrome P450 family 2 subfamily S member 1	1.0925	6.6375	0.000000259	6.075514874	0.745	3.0325	1.23111E-22	4.070469799	2.188788129
Dapp1	dual adaptor of phosphotyrosine and 3-phosphoinositides 1	0.7125	1.0375	0.06385068	1.456140351	0.2175	0.5275	1.3858E-05	2.425287356	1.966824645
Defb36	defensin beta 36	0.4475	1.805	0.02280179	4.033519553	0.1575	1.0375	0.002590506	6.587301587	1.739759036
Dhfr	dihydrofolate reductase	8.015	19.195	0.001974578	2.394884591	6.1825	12.515	0.005300835	2.02426203	1.533759489
Dlx3	distal-less homeobox 3	0.7175	0.1875	0.000000612	0.261324042	1.295	0.2925	2.92506E-08	0.225868726	0.641025641
Dpp4	dipeptidyl peptidase 4	0.94	0.81	0.701997318	0.861702128	0.695	1.715	1.15341E-05	2.467625899	0.472303207
Ecel1	endothelin converting enzyme like 1	0.87	5.8325	1.17E-11	6.704022989	0.2625	1.6325	7.83657E-08	6.219047619	3.572741194
Emp1	epithelial membrane protein 1	14.9375	18.0825	0.554721934	1.210543933	12.2875	33.14	1.65058E-07	2.697049847	0.54563971
En1	engrailed homeobox 1	0.07	1.44	4.75E-15	20.57142857	0	0.5675	8.32556E-10	#DIV/0!	2.537444934
Eqtn	equatorin	0.39	1.33	0.000316747	3.41025641	0.565	0.6525	0.866043451	1.154867257	2.038314176
Eya1	EYA transcriptional coactivator and phosphatase 1	1.23	0.735	0.121229113	0.597560976	0.7675	1.82	0.000174214	2.371335505	0.403846154
Fam240b	family with sequence similarity 240 member B	0.2925	0.625	NA	2.136752137	0.2875	1.04	0.031220103	3.617391304	0.600961538
Fbln1	fibulin 1	8.51	6.7	0.675001704	0.787309048	6.775	14.1925	0.003733037	2.094833948	0.472080324
Fcgr1a	Fc fragment of IgG receptor Ia	2.9475	3.9775	0.612398373	1.349448685	0.895	2.5525	2.27788E-10	2.851955307	1.5582762
Fcgr2b	Fc fragment of IgG receptor IIb	0.7675	1.755	1.02E-10	2.286644951	0.2025	0.86	7.08489E-16	4.24691358	2.040697674
Fcgr3a	Fc fragment of IgG receptor IIIa	0.9375	2.4175	0.005381739	2.578666667	0.18	1.135	1.50552E-07	6.305555556	2.129955947
Fgf2	fibroblast growth factor 2	3.27	11.725	9.95E-17	3.585626911	1.68	5.4325	2.39584E-12	3.233630952	2.158306489
Fgf5	fibroblast growth factor 5	0.405	0.985	0.008342801	2.432098765	0.64	1.93	0.000141239	3.015625	0.510362694
Flrt3	fibronectin leucine rich transmembrane protein 3	7.295	32.66	1.63E-18	4.477039068	5.4525	17.6925	2.42823E-21	3.244841816	1.845979935
Fn1	fibronectin 1	16.1775	17.655	0.860097912	1.091330552	13.2275	26.9725	0.000671128	2.039123039	0.654555566
Foxc2	forkhead box C2	1.975	1.47	0.620865225	0.744303797	1.435	2.8775	0.000719819	2.005226481	0.510860122
Foxd2	forkhead box D2	1.2875	1.4025	0.904945416	1.089320388	1.2625	2.655	0.00235742	2.102970297	0.528248588
Fxyd5	FXYD domain containing ion transport regulator 5	13.175	28.095	4.07E-09	2.132447818	8.125	15.89	5.97411E-11	1.955692308	1.76809314
Gabra5	gamma-aminobutyric acid type A receptor subunit alpha5	3.5175	9.795	1.51E-19	2.784648188	3.89	5.3275	0.235919534	1.369537275	1.83857344
Gadd45a	growth arrest and DNA damage inducible alpha	35.1975	102.8225	3.95E-11	2.921301229	18.72	42.5425	7.59018E-09	2.272569444	2.416936005
Gal	galanin and GMAP prepropeptide	9.56	144.5625	1.92E-46	15.12160042	6.08	85.5475	7.59005E-22	14.0703125	1.689850668
Gcgr	glucagon receptor	0.6225	1.38	0.000469018	2.21686747	1.1	4.355	9.57745E-17	3.959090909	0.316877153
Gjb2	gap junction protein beta 2	2.1775	1.56	0.675537997	0.71641791	3.4475	7.66	0.039272394	2.221899927	0.203655352
Gpr101	G protein-coupled receptor 101	0.0575	0.4375	0.000000897	7.608695652	0.16	2.0025	6.91309E-29	12.515625	0.218476904

Gpr88	G protein-coupled receptor 88	2.4975	5.425	9.17E-15	2.172172172	1.3575	2.6775	7.89011E-07	1.972375691	2.026143791
Grxcr1	glutaredoxin and cysteine rich domain containing 1	0.7725	1.015	0.599429559	1.313915858	0.29	0.6425	0.040790873	2.215517241	1.579766537
Gstm3	glutathione S-transferase mu 3	1.0325	0.16	0.002972843	0.15496368	0.5025	0.5725	0.90351799	1.139303483	0.279475983
Gxylt2	glucoside xylosyltransferase 2	0.7475	0.8	0.937292654	1.070234114	0.7275	1.5775	0.0032998	2.16838488	0.507131537
Hamp	hepcidin antimicrobial peptide	0.365	14.2325	7.59E-08	38.99315068	0.1575	1.34	0.002779515	8.507936508	10.62126866
Hapln2	hyaluronan and proteoglycan link protein	5.1275	2.88	0.0000111	0.561677231	0.7425	0.2325	1.09364E-05	0.313131313	12.38709677
Has1	hyaluronan synthase 1	0.08	1.7825	1.98E-19	22.28125	0.0325	0.59	2.79278E-16	18.15384615	3.021186441
Hcst	hematopoietic cell signal transducer	1.0925	1.1075	0.999893761	1.013729977	0.2325	0.7175	0.039882566	3.086021505	1.543554007
Hpd	4-hydroxyphenylpyruvate dioxygenase	0.08	2.525	0.129612584	31.5625	0.005	0.8075	3.68714E-19	161.5	3.126934985
Icam1	intercellular adhesion molecule 1	2.5875	3.15	0.165146459	1.217391304	1.7775	4.9925	0.001335401	2.808720113	0.63094642
Igf2	insulin like growth factor 2	4.865	2.57	0.399106694	0.528263104	8.1925	18.415	0.03190061	2.247787611	0.139560141
Igsf10	immunoglobulin superfamily member 10	0.2575	0.51	1.53E-10	1.980582524	0.5225	1.43	1.12001E-10	2.736842105	0.356643357
Igsf21	immunoglobin superfamily member 21	3.8925	1.3525	6.08E-13	0.34746307	6.905	2.9	1.91127E-14	0.419985518	0.46637931
Igsf23	immunoglobulin superfamily member 23	0.295	3.665	4.82E-17	12.42372881	0.14	1.4425	1.57092E-11	10.30357143	2.540727903
Igsf6	immunoglobulin superfamily member 6	1.015	1.075	NA	1.0591133	0.275	0.6825	0.002941255	2.481818182	1.575091575
Il24	interleukin 24	0.3525	4.5625	2.21E-17	12.94326241	0.0425	2.075	1.6482E-36	48.82352941	2.198795181
Il6	interleukin 6	0.195	1.685	6.89E-13	8.641025641	0.0525	0.46	0.000206971	8.761904762	3.663043478
Inmt	indolethylamine N-methyltransferase	2.865	4.4275	0.008723295	1.545375218	0.7025	1.5325	0.007837568	2.181494662	2.889070147
Itgb2	integrin subunit beta 2	1.1475	1.7675	0.129043142	1.540305011	0.46	1.1575	1.36086E-09	2.516304348	1.52699784
Lat2	linker for activation of T cells family member 2	1.775	2.6625	0.245358442	1.5	0.5425	1.5575	0.000137109	2.870967742	1.709470305
Lce1f	late cornified envelope 1C	0.2575	15.6975	1.75E-46	60.96116505	0.1175	4.2375	1.6315E-21	36.06382979	3.704424779
Lilrb3	leukocyte immunoglobulin like receptor	1.32	1.4675	0.874801022	1.111742424	0.275	0.8575	0.004340334	3.118181818	1.711370262
LOC100134871	hemoglobin subunit beta-2	6.3425	0.96	0.029733813	0.151359874	46.1325	38.0625	0.675906881	0.825069094	0.025221675
LOC100910270	placenta associated 8	2.8175	0.6825	NA	0.242236025	0.6975	1.46	0.036077817	2.093189964	0.467465753
LOC100910575	serine-threonine kinase receptor-associated protein-like	1.19	13.975	2.66E-08	11.74369748	9.7325	1.24	0.084358912	0.127408169	11.27016129
LOC103694855	hemoglobin subunit beta-2-like	2.49	0.975	0.001786478	0.391566265	6.2025	5.05	0.667519209	0.814187827	0.193069307
LOC299282	serpin family A member 3	0.135	1.6875	0.000000354	12.5	0.025	0.1975	5.98131E-15	7.9	8.544303797
LOC688459	chromosome 6 open reading frame 141	0.6625	4.245	3.01E-32	6.40754717	0.5125	1.525	3.51372E-07	2.975609756	2.783606557
Lox	lysyl oxidase	3.3525	5.455	0.085982157	1.627143922	4.3	10.7575	0.000993307	2.501744186	0.507088078
Loxl1	lysyl oxidase like 1	2.71	3.02	0.861975177	1.114391144	2.31	5.785	0.000396322	2.504329004	0.522039758
Lsmem1	leucine rich single-pass membrane protein 1	0.0275	1.005	5.52E-27	36.54545455	0.01	0.335	4.6547E-13	33.5	3
Ly49si1	immunoreceptor Ly49si2	1.1675	1.0125	0.69756167	0.867237687	0.14	0.4425	0.001110052	3.160714286	2.288135593
Ly6g6c	lymphocyte antigen 6 family member	2.55	3.35	0.467574303	1.31372549	2.2175	5.1925	0.000422681	2.341600902	0.64516129
Lypd1	LY6/PLAUR domain containing 1	6.835	2.795	7.9E-12	0.408924653	6.3575	5.5075	0.00631414	0.866299646	0.507489787
Lyz2	lysozyme	101.9275	160.9	0.37270604	1.578573005	30.2825	72.6825	0.001183701	2.400148601	2.213737832
Mir125b1	microRNA 100	0.835	0.56	NA	0.670658683	0.4025	2.3725	0.034585152	5.894409938	0.236037935
Mir675	microRNA 675	8.67	11.75	0.415561932	1.355247982	50.955	113.3775	0.000105837	2.225051516	0.103636083
Misp3	MISP family member 3	3.085	10.655	8.92E-17	3.453808752	2.9025	4.565	0.029966137	1.572782084	2.334063527
Mmd	monocyte to macrophage differentiation associated	13.175	28.2125	8.31E-31	2.141366224	11.145	18.505	2.33653E-13	1.660385823	1.524587949
Mmp3	matrix metallopeptidase 3	0.1975	1.8875	0.023074655	9.556962025	0.025	0.32	0.001202693	12.8	5.8984375
Ms4a6a	membrane spanning 4-domains A6A	1.6975	2.7825	0.480261524	1.639175258	0.3075	0.765	0.000851583	2.487804878	3.637254902
Nhp2	NHP2 ribonucleoprotein	0.4075	1.4025	0.018669575	3.441717791	0.18	0.29	0.500938245	1.611111111	4.836206897
Nos1	nitric oxide synthase 1	1.0275	3.355	0.00000139	3.265206813	0.6575	1.56	4.78093E-14	2.372623574	2.150641026
Nps	neuropeptide S	0.2925	2.9975	0.000000437	10.24786325	0.2425	0.6	0.273055266	2.474226804	4.995833333
Npy	neuropeptide Y	0.4225	7.8475	5.19E-21	18.5739645	0.2075	2.4275	5.69956E-16	11.69879518	3.232749743
Nts	neurotensin	0.635	1.5775	0.0000459	2.484251969	0.8425	3.7275	4.56484E-11	4.424332344	0.423205902
Osr1	odd-skipped related transcription factor 1	1.035	0.775	0.78995334	0.748792271	1.065	2.245	0.005727768	2.107981221	0.345211581
P2ry6	pyrimidinergic receptor P2Y6	0.88	1.71	0.002341078	1.943181818	0.315	0.8475	3.03358E-06	2.69047619	2.017699115
Padi3	peptidyl arginine deiminase 3	0.4225	1.76	3.98E-16	4.165680473	0.355	0.7475	0.002256069	2.105633803	2.35451505
Parp3	poly(ADP-ribose) polymerase family member 3	14.6575	26.765	6.7E-12	1.826027631	7.875	16.055	6.00917E-11	2.038730159	1.667081906
Pdyn	prodynorphin	0.12	1.1325	1.32E-13	9.4375	0.085	0.6125	6.74069E-12	7.205882353	1.848979592
Penk	proenkephalin	5.93	2.5975	0.0000013	0.438026981	6.07	3.905	0.003591283	0.643327842	0.665172855
Plppr1	phospholipid phosphatase related 1	0.405	0.9025	0.000417365	2.228395062	1.03	1.53	0.050544309	1.485436893	0.589869281
Prdm6	PR/SET domain 6	1.01	0.755	0.614414072	0.747524752	0.6575	1.5875	0.00979526	2.414448669	0.475590551
Prr15l	proline rich 15 like	2.495	0.8825	0.003206971	0.353707415	2.47	2.6175	0.984448427	1.059716599	0.337153773
Prrx2	paired related homeobox 2	0.8475	0.83	0.970710447	0.979351032	0.7225	1.5425	0.040887745	2.134948097	0.53808752
Ptchd1	patched domain containing 1	0.5975	1.475	7.07E-13	2.468619247	0.915	2.5775	5.06885E-16	2.816939891	0.572259942
Ptprc	protein tyrosine phosphatase receptor type C	2.4475	2.6075	0.946734853	1.065372829	0.71	1.7225	1.37043E-11	2.426056338	1.513788099
Ptprh	protein tyrosine phosphatase receptor type H	0.435	7.33	3.02E-17	16.85057471	0.0875	2.1325	2.74305E-10	24.37142857	3.437280188
PVR	poliovirus receptor	5.4975	13.1675	7.55E-13	2.395179627	4.395	7.8575	5.59505E-07	1.787827076	1.675787464
Rasd1	ras related dexamethasone induced 1	2.6	8.75	8.53E-19	3.365384615	1.52	5.3525	2.10721E-13	3.521381579	1.634750117
Rassf10	Ras association domain family member 10	2.3325	5.285	0.000000102	2.265809218	2.3575	3.26	0.132463735	1.382820785	1.621165644
Reg3a	regenerating islet-derived 3 alpha	0.9575	2.7	0.00000066	2.819843342	1.6375	4.215	1.17336E-08	2.574045802	0.640569395
Reg3b	regenerating family member 3 beta	2.215	51.665	7.23E-56	23.32505643	2.8875	33.1775	3.75458E-12	11.49004329	1.55723005
RF00108		5.2875	13.6225	0.000168212	2.576359338	2.6175	2.225	0.674581487	0.850047755	6.12247191
RF00108		6.6525	15.1375	0.000397297	2.275460353	9.4125	9.8175	0.964833668	1.043027888	1.541889483
RF00600		0.6975	0.7725	NA	1.107526882	0.5225	1.895	0.024435163	3.626794258	0.407651715
Rhoq	ras homolog family member Q	23.43	48.3475	0.00004	2.063486983	17.0175	26.7675	0.000677252	1.572939621	1.80620155
Rrad	RRAD, Ras related glycolysis inhibitor and calcium channel regulator	0.82	2.9175	2.25E-11	3.557926829	0.905	1.7625	1.57227E-05	1.947513812	1.655319149
RT1-Ba	major histocompatibility complex, class II, DQ alpha 1	5.0075	10.245	0.04014153	2.045931103	0.925	3.465	1.06345E-08	3.745945946	2.956709957
RT1-Bb	major histocompatibility complex, class II, DQ beta 1	3.465	7.1625	0.353740631	2.067099567	0.3475	1.425	1.26509E-09	4.100719424	5.026315789
RT1-CE7	major histocompatibility complex, class I, A	3.005	3.4275	0.876355595	1.140599002	0.595	1.765	0.02682784	2.966386555	1.941926346
RT1-Da	major histocompatibility complex, class II, DR alpha	15.575	40.68	5.23E-10	2.61187801	3.8675	18.16	6.22596E-08	4.695539754	2.240088106
RT1-Db1	major histocompatibility complex, class II, DR beta 5	2.83	8.6425	0.000519386	3.053886926	1.66	5.1425	8.88251E-16	3.097891566	1.68060282
RT1-DOa	major histocompatibility complex, class II, DO alpha	0.68	1.8	0.000000437	2.647058824	0.185	0.6275	0.001195641	3.391891892	2.868525896
RT1-DOb	major histocompatibility complex, class II, DO beta	0.7575	1.675	0.000047	2.211221122	0.1925	0.5275	0.001469461	2.74025974	3.17535545
RT1-T24-1	histocompatibility 2, T region locus 24	1.255	0.84	0.435420504	0.669322709	0.685	1.4175	0.000741089	2.069343066	0.592592593
Scara3	scavenger receptor class A member 3	7.4775	7.8825	0.886518423	1.054162487	6.1725	13.0725	0.000247458	2.117861482	0.602983362
Scube2	signal peptide, CUB domain and EGF like domain containing 2	0.4325	1.43	9.26E-16	3.306358382	0.3175	0.655	0.000923583	2.062992126	2.183206107
Sdcbp2	syndecan binding protein 2	0.655	1.5525	0.0000338	2.370229008	0.46	0.7025	0.208185249	1.527173913	2.209964413
Selplg	selectin P ligand	2.33	3.45	0.178230188	1.480686695	0.72	1.46	0.028294641	2.027777778	2.363013699
Sema6a	semaphorin 6A	7.195	32.7625	2.18E-20	4.553509382	7.8525	15.9775	8.01808E-24	2.034702324	2.050539822
Serpina1	serpin family A member 1	7.08	15.6575	1.31E-10	2.211511299	3.7425	6.095	2.06863E-07	1.628590514	2.568908942
Serpinb2	serpin family B member 2	0.2025	1.41	2.73E-10	6.962962963	0.0625	0.225	0.008759128	3.6	6.266666667
Serpine1	serpin family E member 1	1.8975	2.7625	0.081115352	1.455862978	0.3875	1.165	2.61768E-05	3.006451613	2.371244635

Shisa3	shisa family member 3	6.71	6.9175	0.986265261	1.030923994	6.3375	13.765	0.028954385	2.17199211	0.502542681
Slc10a4	solute carrier family 10 member 4	1.7175	2.4775	0.214544433	1.442503639	1.7625	0.6675	0.000256826	0.378723404	3.711610487
Slc13a4	solute carrier family 13 member 4	4.5225	2.74	0.432955458	0.605859591	4.6375	9.6425	0.021796329	2.079245283	0.284158673
Slc7a11	solute carrier family 7 member 11	0.505	0.405	0.762103422	0.801980198	0.715	1.78	0.023570419	2.48951049	0.22752809
Slfn4	schlafen family member 4	8.5825	1.285	NA	0.149723274	0.405	2.295	0.024419312	5.666666667	0.559912854
Smagp	small cell adhesion glycoprotein	1.215	7.565	4.62E-21	6.226337449	0.95	4.8325	1.80027E-19	5.086842105	1.565442318
Snai2	snail family transcriptional repressor 2	0.4625	0.84	0.074276935	1.816216216	0.4825	1.34	9.58535E-05	2.777202073	0.626865672
Sox11	SRY-box transcription factor 11	2.41	11.065	7.88E-12	4.591286307	1.9825	6.0325	6.27001E-21	3.042875158	1.834231247
Spata18	spermatogenesis associated 18	0.7925	1.6075	0.000385491	2.028391167	0.7025	0.84	0.5676553	1.195729537	1.913690476
Sprr1a	small proline rich protein 1A	0.5325	12.2275	6.76E-45	22.96244131	0.075	4.4525	7.91686E-51	59.36666667	2.746209994
Stac2	SH3 and cysteine rich domain 2	2.03	28.8475	4.77E-09	14.21059113	1.0425	16.1	3.07363E-18	15.44364508	1.791770186
Steap1	STEAP family member 1	0.6625	1.445	0.000415941	2.181132075	0.545	0.96	0.053200294	1.76146789	1.505208333
Stmn4	stathmin 4	22.705	76.1575	6.7E-11	3.354217133	20.4675	46.535	1.16562E-21	2.273604495	1.636563877
Stra6	signaling receptor and transporter of retinol STRA6	1.015	0.9825	0.956065036	0.967980296	0.6	1.5475	0.000507007	2.579166667	0.634894992
Syt13	synaptotagmin 13	0.7825	1.1	0.220800544	1.405750799	0.95	1.96	1.255E-08	2.063157895	0.56122449
Tagap	T cell activation RhoGTPase activating protein	0.8075	1.07	0.349198088	1.325077399	0.275	0.63	0.006110237	2.290909091	1.698412698
Tdrd12	tudor domain containing 12	0.6375	1.665	NA	2.611764706	0.3525	0.7925	0.000254848	2.24822695	2.100946372
Tgfbi	transforming growth factor beta induced	36.68	54.105	0.055916678	1.475054526	39.3775	85.16	9.28151E-06	2.162656339	0.63533349
Tgm1	transglutaminase 1	0.325	9.8225	1.26E-19	30.22307692	0.1675	4.76	3.50557E-21	28.41791045	2.06355042
Th	tyrosine hydroxylase	0.7075	0.2575	0.003067489	0.363957597	1.4625	0.42	0.000262251	0.287179487	0.613095238
Thbs1	thrombospondin 1	2.8725	4	0.46613786	1.392515231	2.555	8.04	0.001639792	3.146771037	0.497512438
Thbs2	thrombospondin 2	3.2375	3.7525	0.457675441	1.159073359	3.44	11.625	3.23887E-07	3.379360465	0.322795699
Tlr8	toll like receptor 8	0.7275	1.195	0.104598689	1.642611684	0.265	0.7375	0.003687906	2.783018868	1.620338983
Tnik	TRAF2 and NCK interacting kinase	10.555	31.4975	5.48E-15	2.984130744	11.6375	18.71	8.29857E-27	1.60773362	1.683458044
Tph2	tryptophan hydroxylase 2	4.8875	13.67	2.6E-34	2.796930946	4.7825	7.085	5.07397E-07	1.48144276	1.92942837
Tppp2	tubulin polymerization promoting protein family member 2	0.565	1.7025	0.006772353	3.013274336	0.4175	0.8825	0.051323119	2.113772455	1.92917847
Trim15	tripartite motif containing 15	0.28	1.25	0.000190035	4.464285714	0.1575	0.4125	0.00875953	2.619047619	3.03030303
Tslp	thymic stromal lymphopoietin	2.085	19.25	2.55E-34	9.232613909	1.4775	5.505	3.07558E-11	3.725888325	3.496821072
Tspo	translocator protein	12.1825	28.875	0.000137251	2.37020316	5.79	14.4775	3.17652E-11	2.500431779	1.994474184
Ucma	upper zone of growth plate and cartilage matrix associated	0.875	2.015	0.037429852	2.302857143	0.2475	1.0075	0.005440726	4.070707071	2
Ucn	urocortin	0.8125	4.8225	3.45E-13	5.935384615	0.205	2.16	7.45439E-08	10.53658537	2.232638889
Vegfd	vascular endothelial growth factor D	0.4025	0.7625	0.198812013	1.894409938	0.355	1.4325	0.007954682	4.035211268	0.532286213
Vgf	VGF nerve growth factor inducible	5.8975	15.0425	2.13E-22	2.550657058	5.555	10.0025	1.35496E-06	1.800630063	1.503874031
Vwc2	von Willebrand factor C domain containing 2	0.46	1.12	0.00000125	2.434782609	0.53	0.665	0.380791394	1.254716981	1.684210526
Xcl1	X-C motif chemokine ligand 1	0.43	2.215	0.007024595	5.151162791	0.0625	1.2975	7.83668E-09	20.76	1.707129094

Table S3
Genes involved in immunological diseases

Gene Symbol	Entrez Gene Name	FPKM (Adult SNI) /FPKM (Infant SNI)	FPKM (Adult SNI)	FPKM (Infant SNI)
SERPINA3	serpin family A member 3	8.525252525	1.688	0.198
SERPINB2	serpin family B member 2	6.266666667	1.41	0.225
MMP3	matrix metallopeptidase 3	5.9	1.888	0.32
HLA-DQB1	major histocompatibility complex, class II, DQ beta 1	5.025964912	7.162	1.425
NPS	neuropeptide S	4.995	2.997	0.6
IL6	interleukin 6	3.663043478	1.685	0.46
MS4A6A	membrane spanning 4-domains A6A	3.636601307	2.782	0.765
ECEL1	endothelin converting enzyme like 1	3.574142157	5.833	1.632
TSLP	thymic stromal lymphopoietin	3.496821072	19.25	5.505
PTPRH	protein tyrosine phosphatase receptor type H	3.436474449	7.33	2.133
CLEC7A	C-type lectin domain containing 7A	3.360655738	5.74	1.708
NPY	neuropeptide Y	3.232289951	7.848	2.428
HLA-DOB	major histocompatibility complex, class II, DO beta	3.178368121	1.675	0.527
TRIM15	tripartite motif containing 15	3.026634383	1.25	0.413
HAS1	hyaluronan synthase 1	3.022033898	1.783	0.59
HLA-DQA1	major histocompatibility complex, class II, DQ alpha 1	2.956709957	10.245	3.465
HLA-DOA	major histocompatibility complex, class II, DO alpha	2.866242038	1.8	0.628
CIITA	class II major histocompatibility complex transactivator	2.668918919	1.58	0.592
SERPINA1	serpin family A member 1	2.568826907	15.657	6.095
ADGRD1	adhesion G protein-coupled receptor D1	2.457757296	3.2	1.302
GADD45A	growth arrest and DNA damage inducible alpha	2.416976165	102.823	42.542
ATF3	activating transcription factor 3	2.404630811	65.947	27.425
SELPLG	selectin P ligand	2.363013699	3.45	1.46
PADI3	peptidyl arginine deiminase 3	2.352941176	1.76	0.748
HLA-DRA	major histocompatibility complex, class II, DR alpha	2.240088106	40.68	18.16
LYZ	lysozyme	2.213722604	160.9	72.683
CIDEA	cell death inducing DFFA like effector a	2.20201005	6.573	2.985
CYP2S1	cytochrome P450 family 2 subfamily S member 1	2.188262446	6.637	3.033
SCUBE2	signal peptide, CUB domain and EGF like domain containing 2	2.183206107	1.43	0.655
FGF2	fibroblast growth factor 2	2.158107859	11.725	5.433
NOS1	nitric oxide synthase 1	2.150641026	3.355	1.56
FCGR3A/FCGR3B	Fc fragment of IgG receptor IIIa	2.129515419	2.417	1.135
CD74	CD74 molecule	2.126113665	261.31	122.905
FCGR2B	Fc fragment of IgG receptor IIb	2.040697674	1.755	0.86
TSPO	translocator protein	1.994543068	28.875	14.477
CSF1	colony stimulating factor 1	1.955609756	12.027	6.15
HLA-A	major histocompatibility complex, class I, A	1.942209632	3.428	1.765
C1QA	complement C1q A chain	1.928015784	34.203	17.74
CCKBR	cholecystokinin B receptor	1.906470588	6.482	3.4
PDYN	prodynorphin	1.846655791	1.132	0.613
GABRA5	gamma-aminobutyric acid type A receptor subunit alpha5	1.838400901	9.795	5.328
SOX11	SRY-box transcription factor 11	1.834383289	11.065	6.032
CTSS	cathepsin S	1.828476621	30.307	16.575
C1QC	complement C1q C chain	1.786083362	25.925	14.515
CLEC12A	C-type lectin domain family 12 member A	1.776098901	6.465	3.64
FXYD5	FXYD domain containing ion transport regulator 5	1.76809314	28.095	15.89
CD86	CD86 molecule	1.716666667	1.133	0.66
LAT2	linker for activation of T cells family member 2	1.710340398	2.663	1.557
XCL1	X-C motif chemokine ligand 1	1.707787201	2.215	1.297
TAGAP	T cell activation RhoGTPase activating protein	1.698412698	1.07	0.63
HLA-DRB5	major histocompatibility complex, class II, DR beta 5	1.680536652	8.643	5.143
Pvr	poliovirus receptor	1.675957745	13.168	7.857
CD68	CD68 molecule	1.621301775	4.11	2.535
RASSF10	Ras association domain family member 10	1.621165644	5.285	3.26
Clec4a3	C-type lectin domain family 4, member a3	1.619838524	7.022	4.335
TLR8	toll like receptor 8	1.619241192	1.195	0.738
GRXCR1	glutaredoxin and cysteine rich domain containing 1	1.580996885	1.015	0.642
CCR5	C-C motif chemokine receptor 5	1.577039275	2.61	1.655
CCDC172	coiled-coil domain containing 172	1.56056209	10.217	6.547
FCGR1A	Fc fragment of IgG receptor Ia	1.558166863	3.978	2.553
HCST	hematopoietic cell signal transducer	1.543175487	1.108	0.718
DHFR	dihydrofolate reductase	1.533759489	19.195	12.515
ITGB2	integrin subunit beta 2	1.527225583	1.767	1.157
MMD	monocyte to macrophage differentiation associated	1.524560929	28.212	18.505
PTPRC	protein tyrosine phosphatase receptor type C	1.513058619	2.607	1.723
STEAP1	STEAP family member 1	1.505208333	1.445	0.96
FN1	fibronectin 1	0.654543432	17.655	26.973
LY6G6C	lymphocyte antigen 6 family member G6C	0.645223421	3.35	5.192
TGFBI	transforming growth factor beta induced	0.63533349	54.105	85.16
ICAM1	intercellular adhesion molecule 1	0.631009615	3.15	4.992
SNAI2	snail family transcriptional repressor 2	0.626865672	0.84	1.34
CCL11	C-C motif chemokine ligand 11	0.615740741	0.665	1.08
PTCHD1	patched domain containing 1	0.572370974	1.475	2.577
COL8A1	collagen type VIII alpha 1 chain	0.563587003	6.643	11.787
BTC	betacellulin	0.563418617	1.035	1.837
SYT13	synaptotagmin 13	0.56122449	1.1	1.96

EMP1	epithelial membrane protein 1	0.545624623	18.082	33.14
VEGFD	vascular endothelial growth factor D	0.53175157	0.762	1.433
LOXL1	lysyl oxidase like 1	0.522039758	3.02	5.785
THBS1	thrombospondin 1	0.497512438	4	8.04
CCND2	cyclin D2	0.475765926	5.87	12.338
CRYBG1	crystallin beta-gamma domain containing 1	0.444141689	2.445	5.505
NTS	neurotensin	0.423015021	1.577	3.728
THBS2	thrombospondin 2	0.322752688	3.752	11.625
GCGR	glucagon receptor	0.316877153	1.38	4.355
CXCL14	C-X-C motif chemokine ligand 14	0.316687898	1.243	3.925
GPR101	G protein-coupled receptor 101	0.218671992	0.438	2.003

Table S4
Cytokines involved in immunological diseases or inflammatory response

Gene Symbol	Entrez Gene Name	FPKM (Adult SNI) /FPKM (Infant SNI)	FPKM (Adult SNI)	FPKM (Infant SNI)
IL6	interleukin 6	3.663043478	1.685	0.46
TSLP	thymic stromal lymphopoietin	3.496821072	19.25	5.505
IL24	interleukin 24	2.198554217	4.562	2.075
CSF1	colony stimulating factor 1	1.955609756	12.027	6.15
XCL1	X-C motif chemokine ligand 1	1.707787201	2.215	1.297
CCL11	C-C motif chemokine ligand 11	0.615740741	0.665	1.08
CXCL14	C-X-C motif chemokine ligand 14	0.316687898	1.243	3.925

Table S5
Genes involved in inflammatory response

Gene Symbol	Entrez Gene Name	FPKM (Adult SNI) /FPKM (Infant SNI)	FPKM (Adult SNI)	FPKM (Infant SNI)
SERPINA3	serpin family A member 3	8.525252525	1.688	0.198
SERPINB2	serpin family B member 2	6.266666667	1.41	0.225
MMP3	matrix metallopeptidase 3	5.9	1.888	0.32
HLA-DQB1	major histocompatibility complex, class II, DQ beta 1	5.025964912	7.162	1.425
IL6	interleukin 6	3.663043478	1.685	0.46
MS4A6A	membrane spanning 4-domains A6A	3.636601307	2.782	0.765
TSLP	thymic stromal lymphopoietin	3.496821072	19.25	5.505
CLEC7A	C-type lectin domain containing 7A	3.360655738	5.74	1.708
NPY	neuropeptide Y	3.232289951	7.848	2.428
HLA-DOB	major histocompatibility complex, class II, DO beta	3.178368121	1.675	0.527
TRIM15	tripartite motif containing 15	3.026634383	1.25	0.413
HLA-DQA1	major histocompatibility complex, class II, DQ alpha 1	2.956709957	10.245	3.465
HLA-DOA	major histocompatibility complex, class II, DO alpha	2.866242038	1.8	0.628
SPRR1A	small proline rich protein 1A	2.745789355	12.227	4.453
CIITA	class II major histocompatibility complex transactivator	2.668918919	1.58	0.592
SERPINA1	serpin family A member 1	2.568826907	15.657	6.095
GADD45A	growth arrest and DNA damage inducible alpha	2.416976165	102.823	42.542
ATF3	activating transcription factor 3	2.404630811	65.947	27.425
SERPINE1	serpin family E member 1	2.370815451	2.762	1.165
SELPLG	selectin P ligand	2.363013699	3.45	1.46
HLA-DRA	major histocompatibility complex, class II, DR alpha	2.240088106	40.68	18.16
UCN	urocortin	2.232407407	4.822	2.16
LYZ	lysozyme	2.213722604	160.9	72.683
IL24	interleukin 24	2.198554217	4.562	2.075
CYP2S1	cytochrome P450 family 2 subfamily S member 1	2.188262446	6.637	3.033
FGF2	fibroblast growth factor 2	2.158107859	11.725	5.433
NOS1	nitric oxide synthase 1	2.150641026	3.355	1.56
FCGR3A/FCGR3B	Fc fragment of IgG receptor IIIa	2.129515419	2.417	1.135
CD74	CD74 molecule	2.126113665	261.31	122.905
FCGR2B	Fc fragment of IgG receptor IIb	2.040697674	1.755	0.86
TSPO	translocator protein	1.994543068	28.875	14.477
CSF1	colony stimulating factor 1	1.955609756	12.027	6.15
HLA-A	major histocompatibility complex, class I, A	1.942209632	3.428	1.765
C1QA	complement C1q A chain	1.928015784	34.203	17.74
CCKBR	cholecystokinin B receptor	1.906470588	6.482	3.4
PDYN	prodynorphin	1.846655791	1.132	0.613
GABRA5	gamma-aminobutyric acid type A receptor subunit alpha5	1.838400901	9.795	5.328
SOX11	SRY-box transcription factor 11	1.834383289	11.065	6.032
CTSS	cathepsin S	1.828476621	30.307	16.575
CSRP3	cysteine and glycine rich protein 3	1.750130141	16.81	9.605
CD86	CD86 molecule	1.716666667	1.133	0.66
XCL1	X-C motif chemokine ligand 1	1.707787201	2.215	1.297
TAGAP	T cell activation RhoGTPase activating protein	1.698412698	1.07	0.63
GAL	galanin and GMAP prepropeptide	1.6898547	144.562	85.547
HLA-DRB5	major histocompatibility complex, class II, DR beta 5	1.680536652	8.643	5.143
Pvr	poliovirus receptor	1.675957745	13.168	7.857
CD68	CD68 molecule	1.621301775	4.11	2.535
TLR8	toll like receptor 8	1.619241192	1.195	0.738
CCR5	C-C motif chemokine receptor 5	1.577039275	2.61	1.655
FCGR1A	Fc fragment of IgG receptor Ia	1.558166863	3.978	2.553
REG3A	regenerating family member 3 alpha	1.557253519	51.665	33.177
HCST	hematopoietic cell signal transducer	1.543175487	1.108	0.718
DHFR	dihydrofolate reductase	1.533759489	19.195	12.515
ITGB2	integrin subunit beta 2	1.527225583	1.767	1.157
MMD	monocyte to macrophage differentiation associated	1.524560929	28.212	18.505
PTPRC	protein tyrosine phosphatase receptor type C	1.513058619	2.607	1.723
FN1	fibronectin 1	0.654543432	17.655	26.973
LY6G6C	lymphocyte antigen 6 family member G6C	0.645223421	3.35	5.192
TGFBI	transforming growth factor beta induced	0.63533349	54.105	85.16
ICAM1	intercellular adhesion molecule 1	0.631009615	3.15	4.992
SNAI2	snail family transcriptional repressor 2	0.626865672	0.84	1.34
CCL11	C-C motif chemokine ligand 11	0.615740741	0.665	1.08
BTC	betacellulin	0.563418617	1.035	1.837
VEGFD	vascular endothelial growth factor D	0.53175157	0.762	1.433
LOXL1	lysyl oxidase like 1	0.522039758	3.02	5.785
LOX	lysyl oxidase	0.50706451	5.455	10.758
THBS1	thrombospondin 1	0.497512438	4	8.04
THBS2	thrombospondin 2	0.322752688	3.752	11.625
CXCL14	C-X-C motif chemokine ligand 14	0.316687898	1.243	3.925
